# Supplementary material for: Tuning the Biological Activity of PI3Kδ Inhibitor by the Introduction of a Fluorine Atom Using the Computational Workflow
Source: Molecules. 2023 Apr 17;28(8):3531. doi: 10.3390/molecules28083531 (PMC10145010; doi:10.3390/molecules28083531)
Supplement: Supplementary file 1 [file molecules-28-03531-s001.zip › molecules-2260696-supplementary.pdf]

Supplementary data

for

Tuning the Biological Activity of PI3Kδ Inhibitor by the Introduction of a Fluorine Atom Using the Computational Workflow

Wojciech Pietruś [1,2,*], Mariola Stypik [2,3], Marcin Zagozda [2], Martyna Banach [2], Lidia Gurba-Bryśkiewicz [2], Wioleta Maruszak [2], Arkadiusz Leniak [2], Rafał Kurczab [1,*], Zbigniew Ochal [3], Krzysztof Dubiel [2] and Maciej Wieczorek [2]

[1] Department of Medicinal Chemistry, Maj Institute of Pharmacology, Polish Academy of Sciences, Smetna 12, 31-343 Krakow, Poland

[2] Celon Pharma S.A., ul. Marymoncka 15, 05-152 Kazuń Nowy, Poland

[3] Faculty of Chemistry, Warsaw University of Technology, ul. Nowakowskiego 3, 00-664 Warsaw, Poland

* Correspondence: pietrus@if-pan.krakow.pl (W.P.); kurczab@if-pan.krakow.pl (R.K.); Tel.: +48-126-62-3301 (R.K.)

Compound 1 ^{1}H NMR and ^{13}C NMR 3
Compound 1 HRMS 4
Compound 2 ^{1}H NMR and ^{13}C NMR 5
Compound 2 HRMS 6
Compound 3 ^{1}H NMR and ^{13}C NMR 7
Compound 3 HRMS 8
Compound 4 ^{1}H NMR and ^{13}C NMR 9
Compound 4 HRMS 10
Compound 5 ^{1}H NMR and ^{13}C NMR 11
Compound 5 HRMS 12
Compound 6 ^{1}H NMR and ^{13}C NMR 13
Compound 6 HRMS 14
Compound 7 ^{1}H NMR and ^{13}C NMR 15
Compound 7 HRMS 16
Compound 8 ^{1}H NMR and ^{13}C NMR 17
Compound 8 HRMS 18
Compound 9 ^{1}H NMR and ^{13}C NMR 19
Compound 9 HRMS 20
Compound 10 ^{1}H NMR and ^{13}C NMR 21
Compound 10 HRMS 22
Compound 11 ^{1}H NMR and ^{13}C NMR 23
Compound 11 HRMS 24
Compound 12 ^{1}H NMR and ^{13}C NMR 25
Compound 12 HRMS 26
Compound 13 ^{1}H NMR and ^{13}C NMR 27
Compound 13 HRMS 28
Synthesis of 5-(2-difluoromethylobenzimidazo-1-yl)pyrazolo[1,5-a]pyrimidine derivatives. 29
The model system of PI3Kδ with lipid bilayer 30
The results QPLD docking approach with different level of flexibility of protein. 31

Compound 1 ^{1}H NMR and ^{13}C NMR

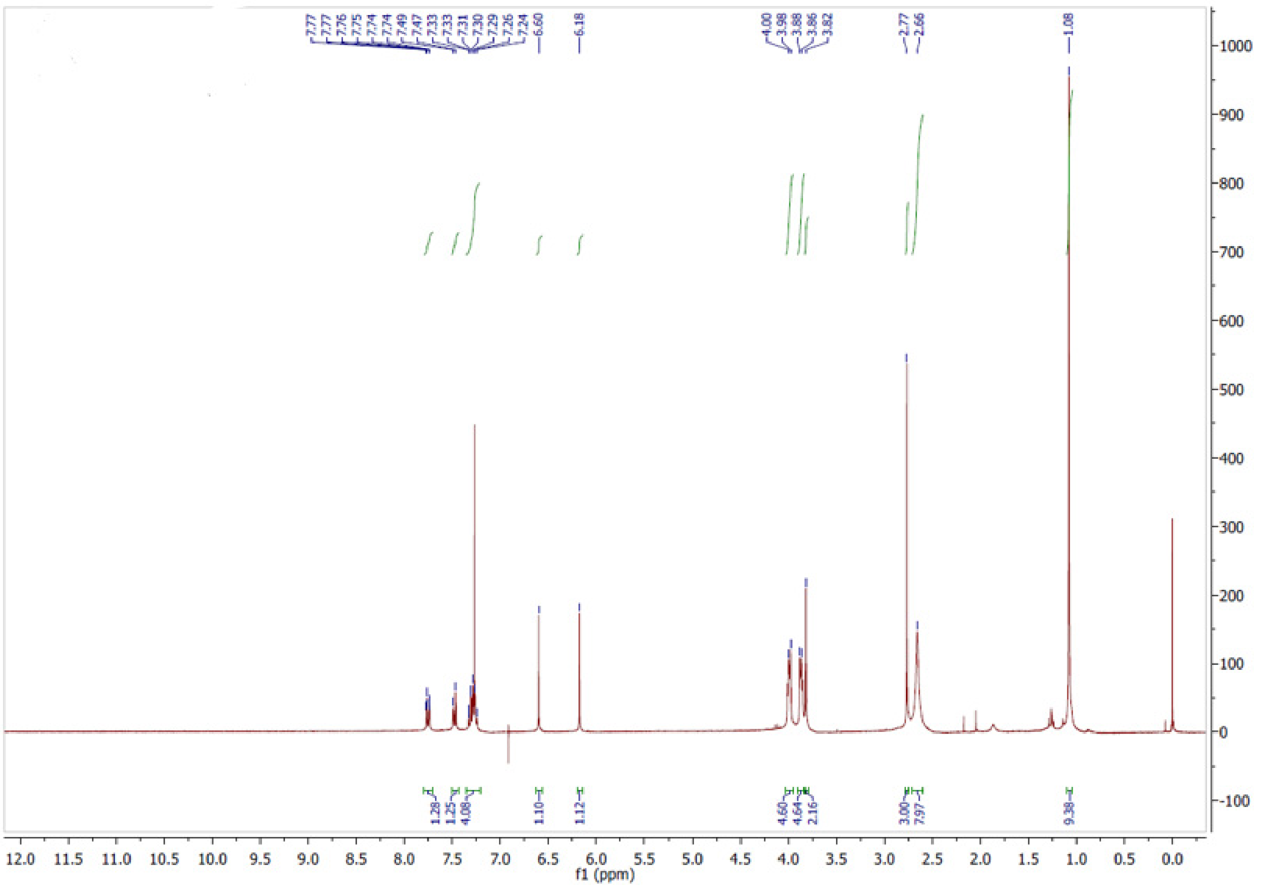

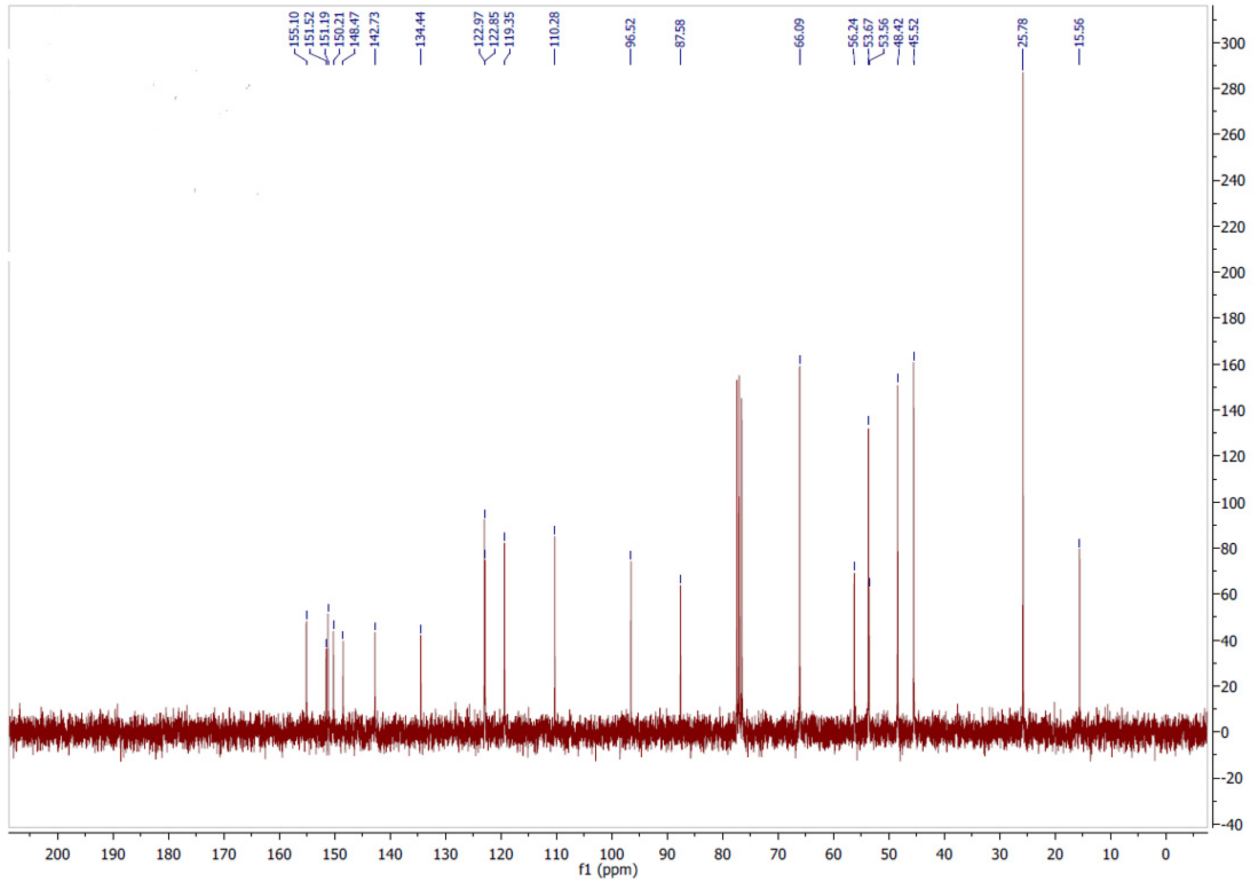

Compound 1 HRMS

Compound Label	Name	*m/z*	RT	Algorithm	Mass
Compound	Compound	489.30884	0.24	Find by Formula	488.30154

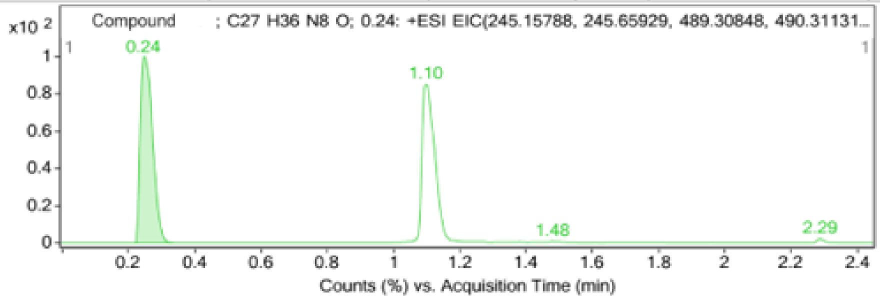

MS Zoomed Spectrum

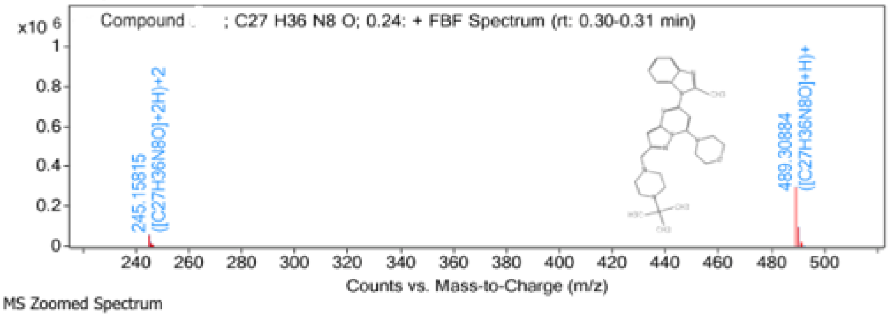

MS Zoomed Spectrum

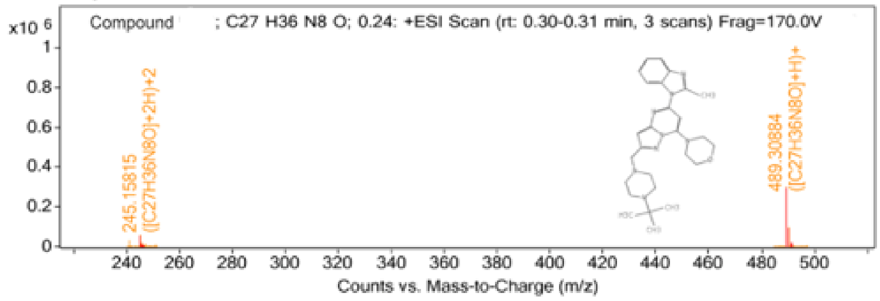

MS Spectrum Peak List

m/z	*Calc m/z*	Diff(ppm)	z	Abund	Formula	Ion
245.15815	245.15788	1.12	2	56190	C27H36N8O	(M+2H)+2
245.65963	245.65929	1.38	2	18164.55	C27H36N8O	(M+2H)+2
246.16121	246.16067	2.2	2	2663.72	C27H36N8O	(M+2H)+2
246.66092	246.66202	-4.46	2	280.06	C27H36N8O	(M+2H)+2
489.30884	489.30848	0.72	1	298324.84	C27H36N8O	(M+H)+
490.31149	490.31131	0.37	1	97178.29	C27H36N8O	(M+H)+
491.31367	491.31407	-0.81	1	14949.13	C27H36N8O	(M+H)+
492.31654	492.31675	-0.42	1	1609.93	C27H36N8O	(M+H)+

--- End Of Report ---

Compound 2 ^{1}H NMR and ^{13}C NMR

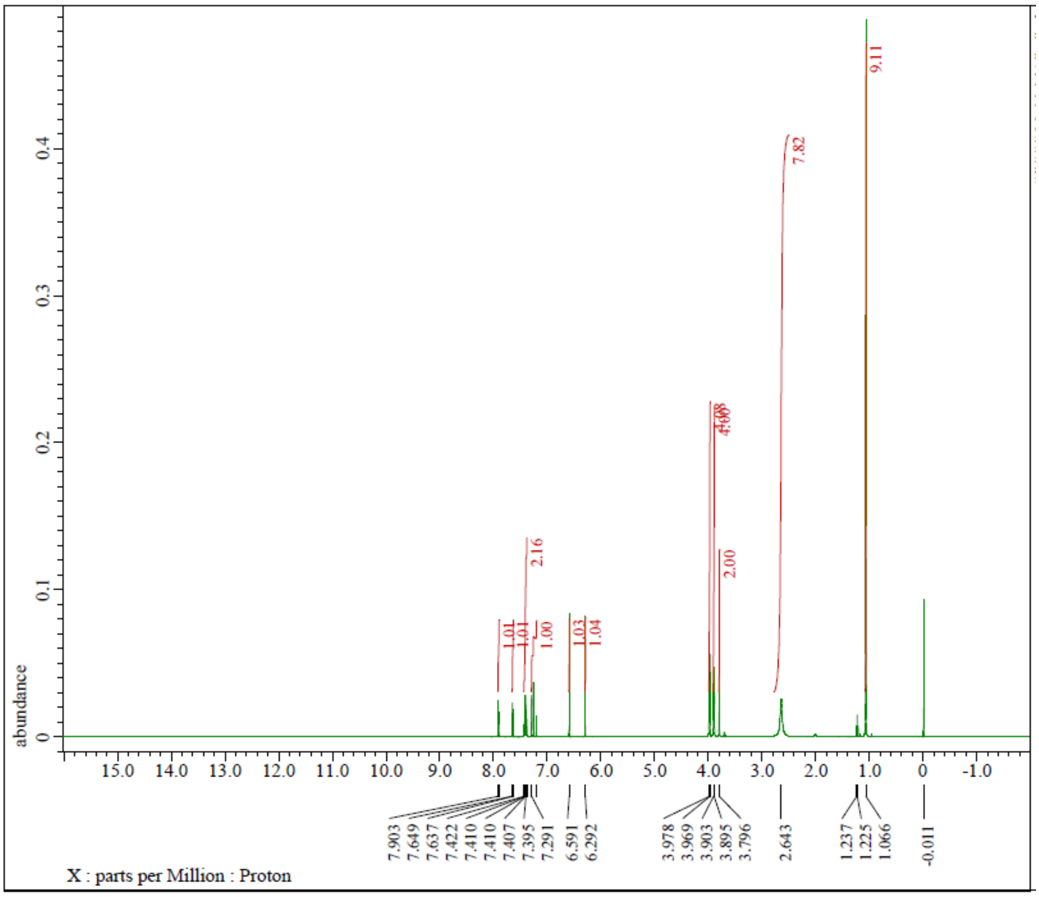

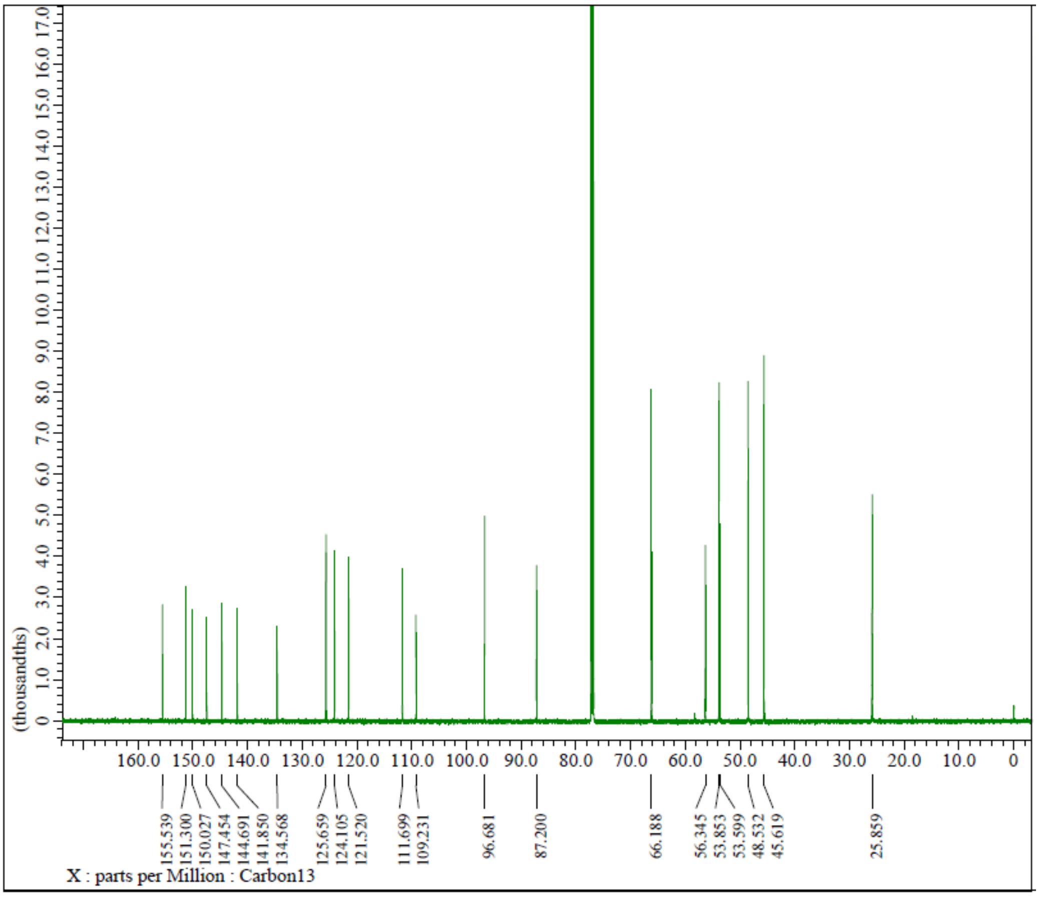

Compound 2 HRMS

Compound Table

Compound Label	RT	Mass	Abund	Name	Formula	Tgt Mass	Diff (ppm)	Hits (DB)
Cpd 2: 302-415-10; C27 H34 F2 N8 O; 2.414	2.414	524.28269	86308		C27 H34 F2 N8 O	524.28236	0.63	1

Compound Label	Name	*m/z*	RT	Algorithm	Mass
Cpd 2: 302-415-10; C27 H34 F2 N8 O; 2.414		525.29001	2.414	Find by Formula	524.28269

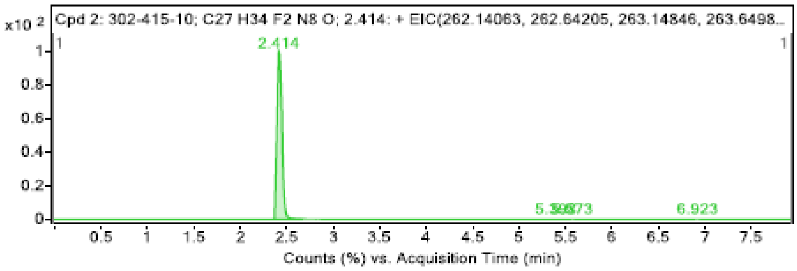

MS Spectrum

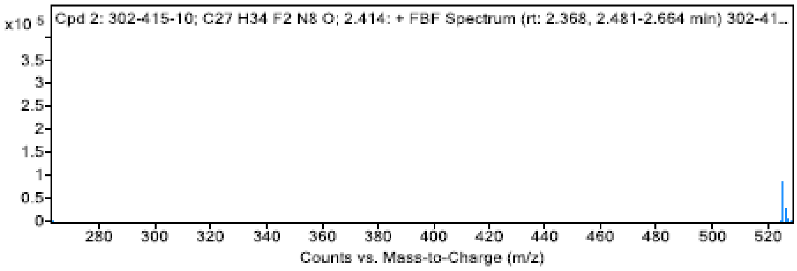

MS Zoomed Spectrum

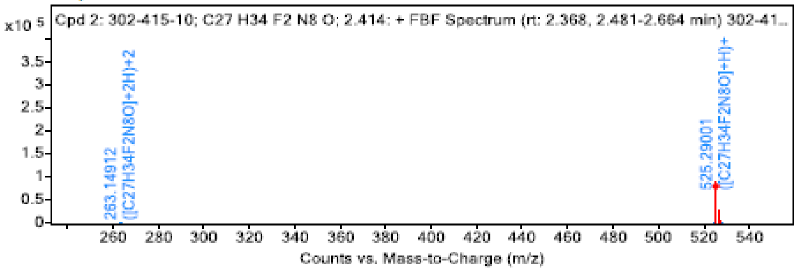

MS Spectrum Peak List

m/z	z	Abund	Formula	Ion
263.14912	2	359.79	C27H34F2N8O	(M+2H)+2
263.64952	2	96.69	C27H34F2N8O	(M+2H)+2
524.27521	1	183.57	C27H34F2N8O	M+
525.29001	1	86308.49	C27H34F2N8O	(M+H)+
526.29271	1	27103.87	C27H34F2N8O	(M+H)+
527.29545	1	3988.21	C27H34F2N8O	(M+H)+
528.29851	1	428.02	C27H34F2N8O	(M+H)+

MS Spectrum

Compound 3 ^{1}H NMR and ^{13}C NMR

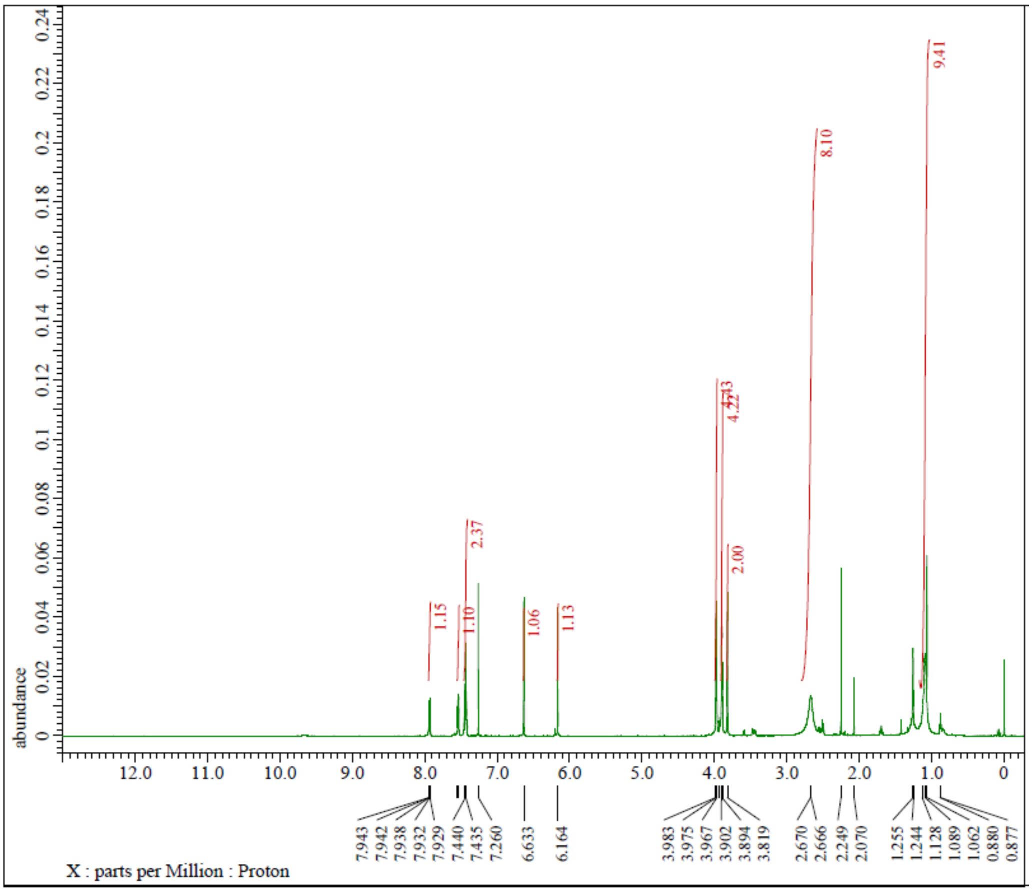

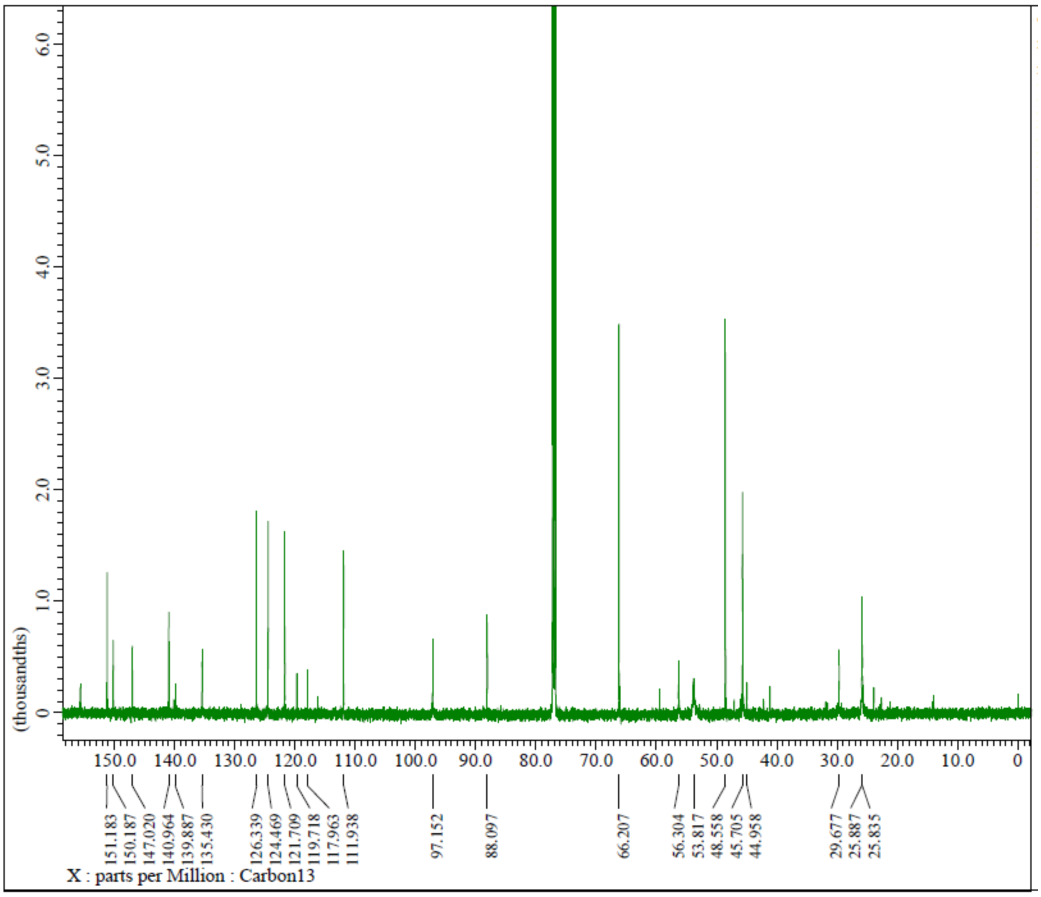

Compound 3 HRMS

Name	*Obs. m/z*	Obs. RT	Obs. Mass	Tgt Formula	Tgt Mass	Tgt Mass Error	Find Cpds Algorit
	272.14376	1.41	542.27339	C27 H33 F3 N8 O	542.27294	0.83	Find by Formula

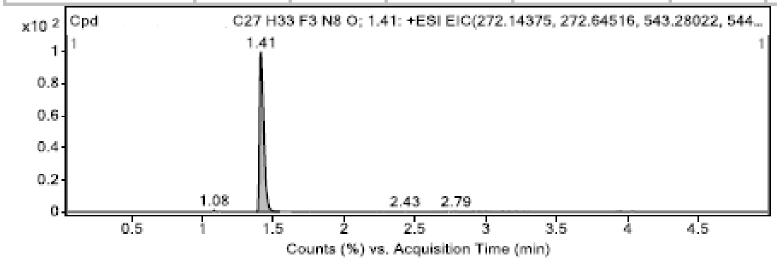

MS Zoomed Spectrum

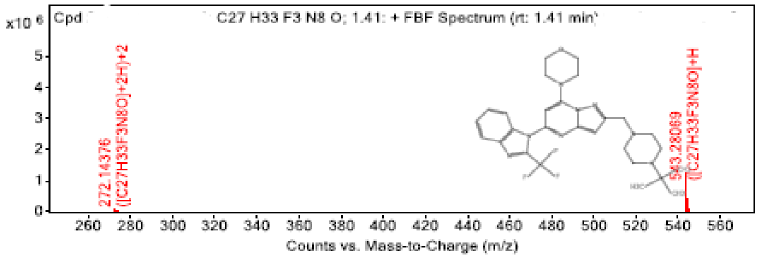

MS Zoomed Spectrum

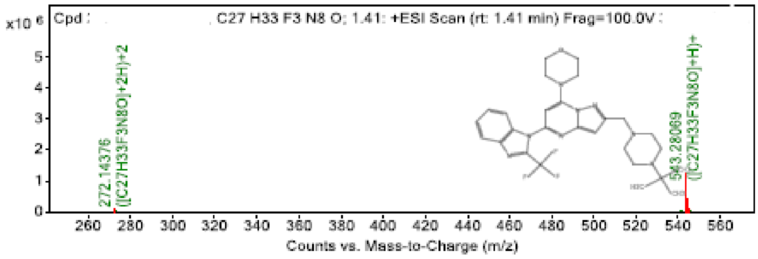

MS Spectrum Peak List

Obs. m/z	Charge	Abund	Formula	Ion/Isotope	Tgt Mass Error (ppm)
272.14376	2	65592.5	C27H33F3N8O	(M+2H)+2	
272.6453	2	23443.74	C27H33F3N8O	(M+2H)+2	
273.14618	2	4575.98	C27H33F3N8O	(M+2H)+2	
273.64635	2	210.29	C27H33F3N8O	(M+2H)+2	
543.28069	1	1223762.88	C27H33F3N8O	(M+H)+	
544.28358	1	331950.72	C27H33F3N8O	(M+H)+	
545.28592	1	53670.24	C27H33F3N8O	(M+H)+	
272.14376	2	65592.5	C27H33F3N8O	(M+2H)+2	0.03
272.6453	2	23443.74	C27H33F3N8O	(M+2H)+2	0.53
273.14618	2	4575.98	C27H33F3N8O	(M+2H)+2	-1.32
273.64635	2	210.29	C27H33F3N8O	(M+2H)+2	-5.59
543.28069	1	1223762.88	C27H33F3N8O	(M+H)+	0.87
544.28358	1	331950.72	C27H33F3N8O	(M+H)+	0.99
545.28592	1	53670.24	C27H33F3N8O	(M+H)+	0.23

--- End Of Report ---

Compound 4 ^{1}H NMR and ^{13}C NMR

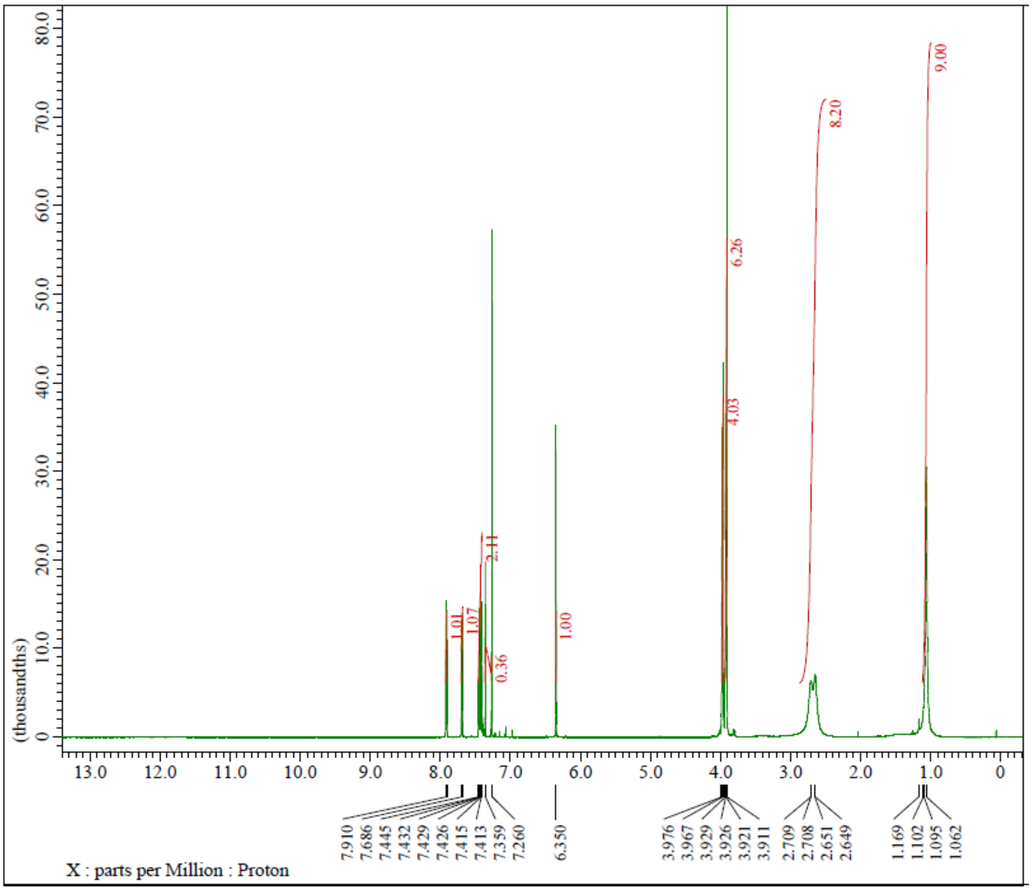

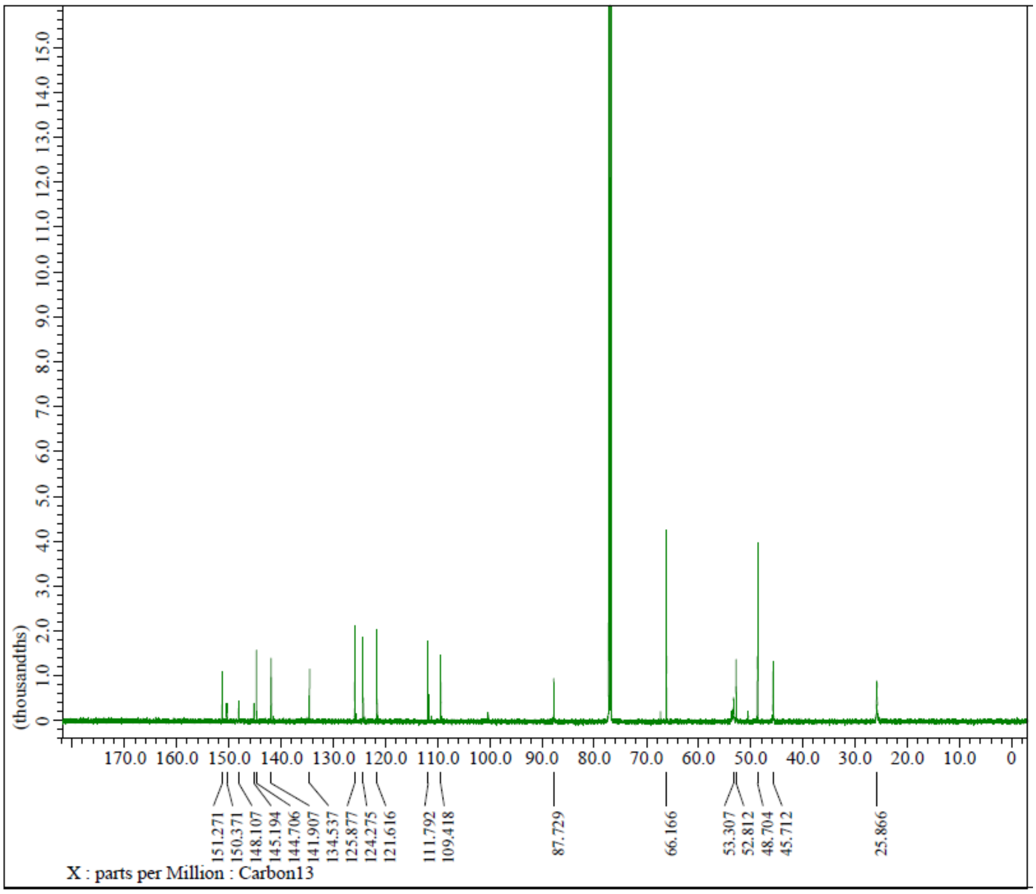

Compound 4 HRMS

Compound Table

Compound Label	RT	Mass	Abund	Name	Formula	Tgt Mass	Diff (ppm)	Hits (DB)
Cpd 3: C27 H33 Cl F2 N8 O; 1.53	1.53	558.24419	23873	302-462-01	C27 H33 Cl F2 N8 O	558.24339	1.43	1

Compound Label	Name	*m/z*	RT	Algorithm	Mass
Cpd 3: C27 H33 Cl F2 N8 O; 1.53		280.12924	1.53	Find by Formula	558.24419

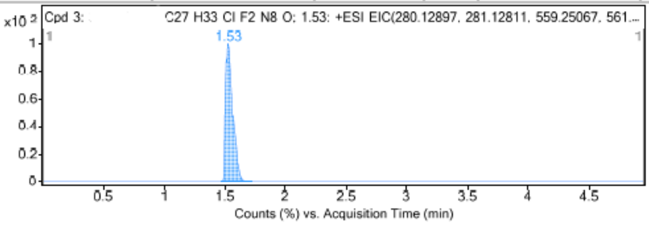

MS Spectrum

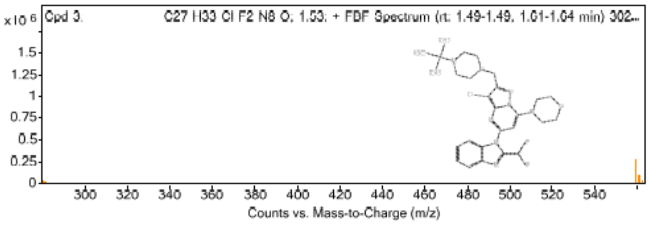

MS Zoomed Spectrum

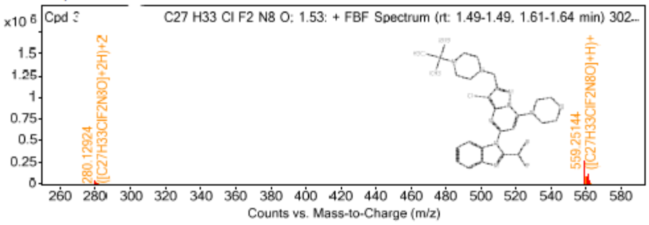

MS Spectrum Peak List

m/z	z	Abund	Formula	Ion
280.12924	2	23873.35	C27H33ClF2N8O	(M+2H)+2
280.63059	2	7657.21	C27H33ClF2N8O	(M+2H)+2
281.12876	2	7946.88	C27H33ClF2N8O	(M+2H)+2
281.62912	2	2427.62	C27H33ClF2N8O	(M+2H)+2
282.1316	2	436.01	C27H33ClF2N8O	(M+2H)+2

559.25144	1	269523.34	C27H33ClF2N8O	(M+H)+
560.25425	1	84915.55	C27H33ClF2N8O	(M+H)+
561.25009	1	90339.77	C27H33ClF2N8O	(M+H)+
562.25139	1	27122.02	C27H33ClF2N8O	(M+H)+
563.25329	1	4049.36	C27H33ClF2N8O	(M+H)+

Compound 5 ^{1}H NMR and ^{13}C NMR

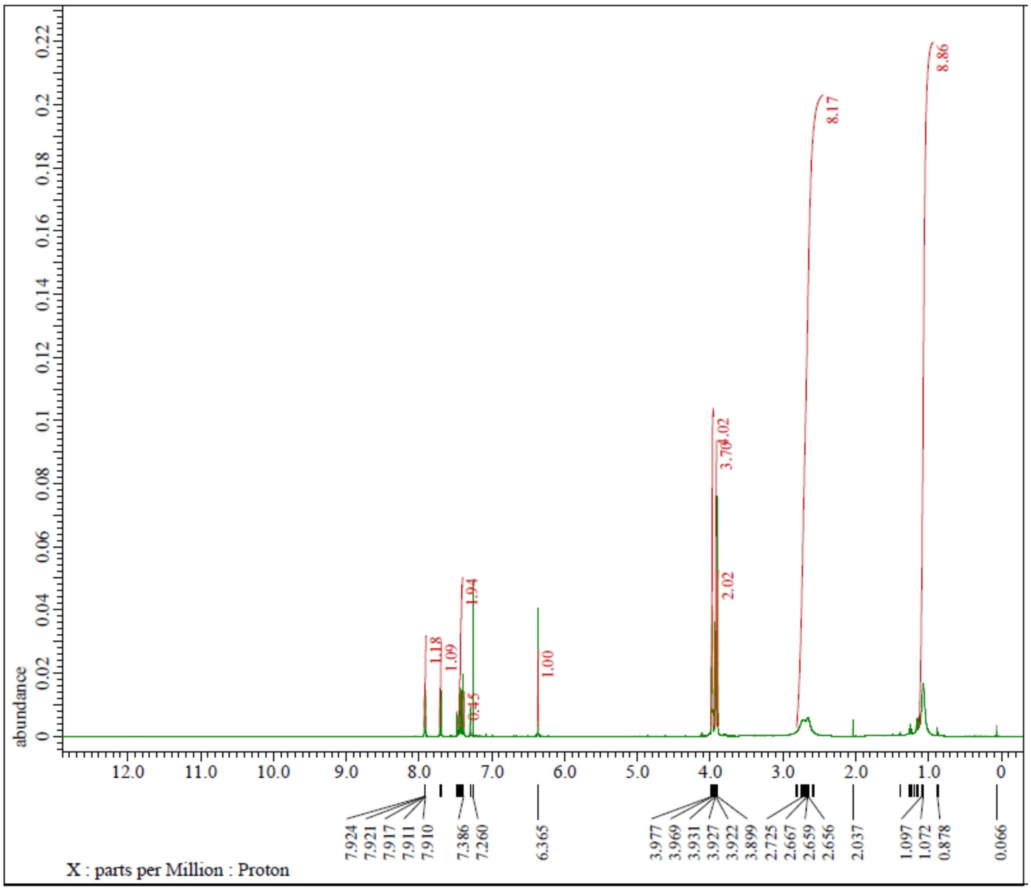

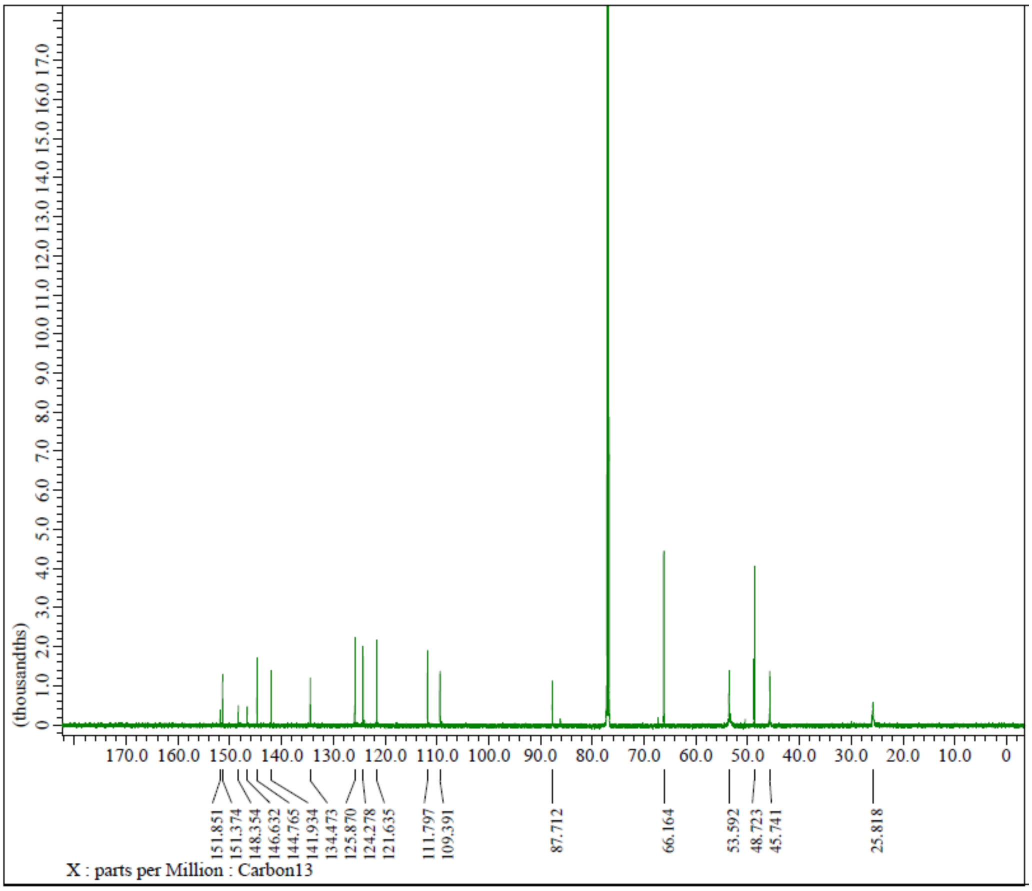

Compound 5 HRMS

Compound Table

Compound Label	RT	Mass	Abund	Name	Formula	Tgt Mass	Diff (ppm)	Hits (DB)
Cpd 4: ; C27 H33 Br F2 N8 O; 1.54	1.54	602.19358	21191	302-472-01	C27 H33 Br F2 N8 O	602.19288	1.16	1

Compound Label	Name	*m/z*	RT	Algorithm	Mass
Cpd 4: ; C27 H33 Br F2 N8 O; 1.54		303.10333	1.54	Find by Formula	602.19358

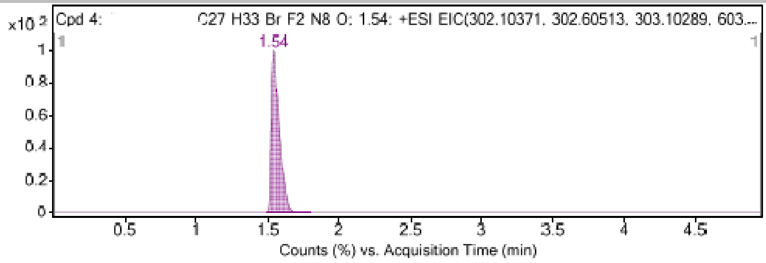

MS Spectrum

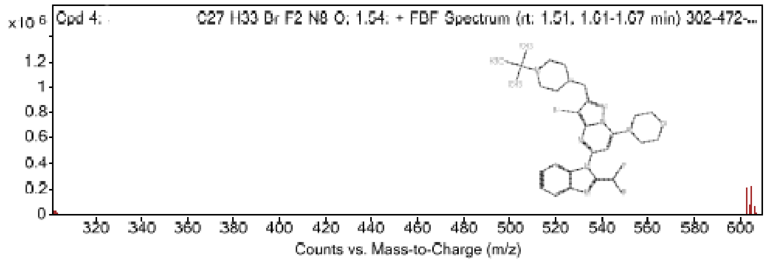

MS Zoomed Spectrum

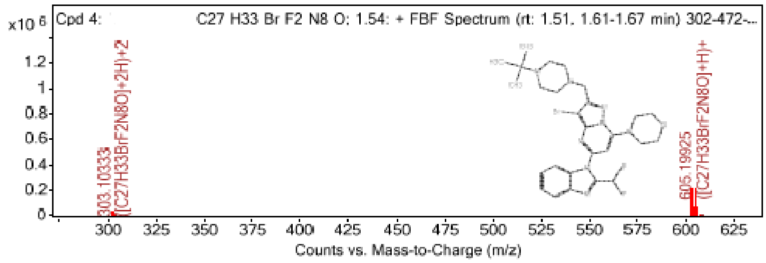

MS Spectrum Peak List

m/z	z	Abund	Formula	Ion
302.10407	2	20756.58	C27H33BrF2N8O	(M+2H)+2
302.60544	2	6840.31	C27H33BrF2N8O	(M+2H)+2
303.10333	2	21191.05	C27H33BrF2N8O	(M+2H)+2
303.60448	2	6691.11	C27H33BrF2N8O	(M+2H)+2
603.20087	1	210262.8	C27H33BrF2N8O	(M+H)+
604.20373	1	67648.03	C27H33BrF2N8O	(M+H)+
605.19925	1	211607.64	C27H33BrF2N8O	(M+H)+
606.20161	1	65050.42	C27H33BrF2N8O	(M+H)+
607.20368	1	9957.62	C27H33BrF2N8O	(M+H)+
608.20635	1	1048.36	C27H33BrF2N8O	(M+H)+

Compound 6 ^{1}H NMR and ^{13}C NMR

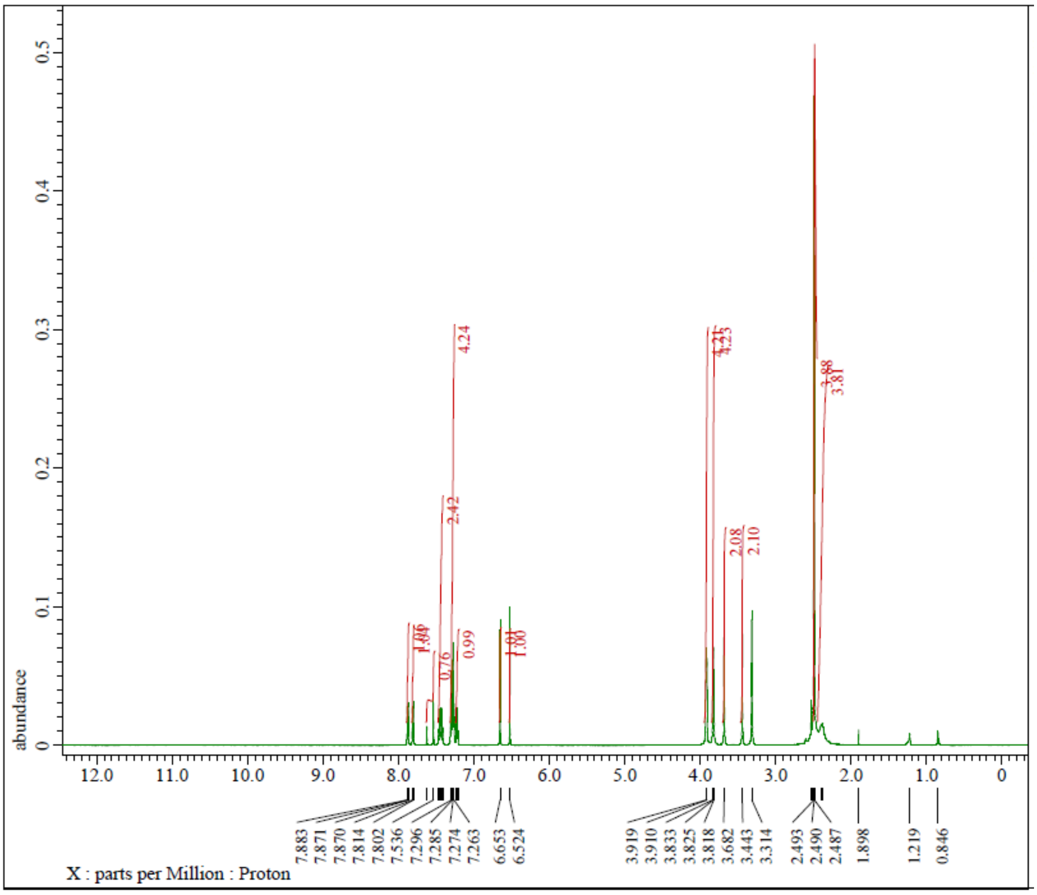

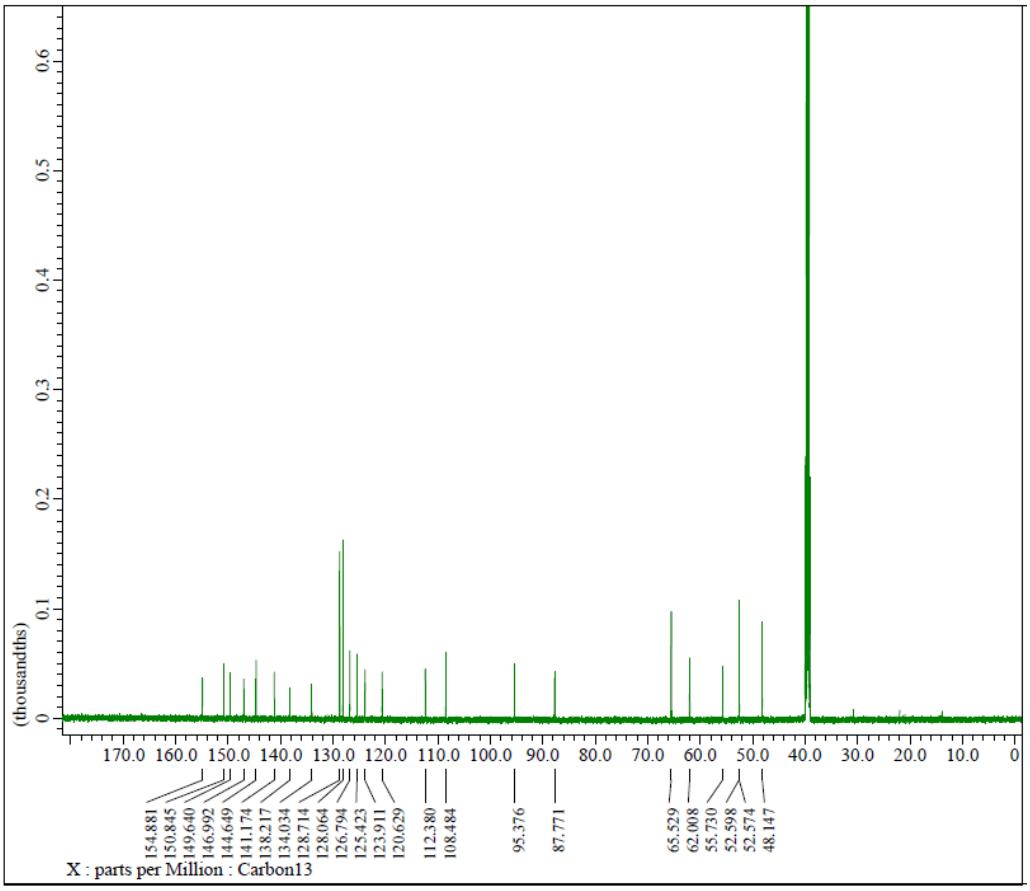

Compound 6 HRMS

Name	*Obs. m/z*	Obs. RT	Obs. Mass	DB RT	DB Formula	DB Mass	DB Mass Error	Tgt RT	Tgt Formula	Tgt Mass	Tgt Mass Error	RT Diff.	Find Cpds Algorith
	559.2754	1.25	558.2681						C30 H32 F2 N8 O	558.2667	2.44		Find by Formula

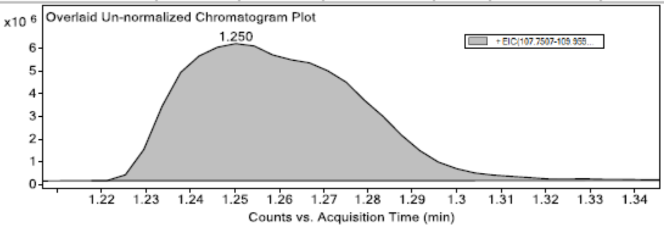

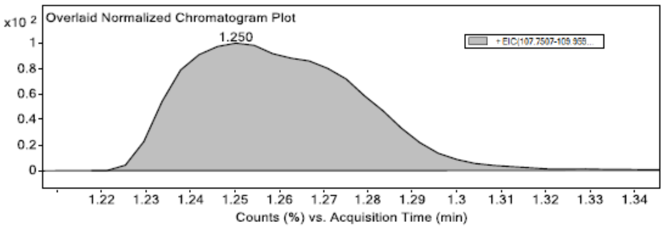

MS Spectrum Peak List

Obs. m/z	Charge	Abund	Formula	Ion/Isotope	Tgt Mass Error (ppm)
280.1427	2	1530.98	C30H32F2N8O	(M+2H)+2	
280.6426	2	558.26	C30H32F2N8O	(M+2H)+2	
558.2623	1	438.14	C30H32F2N8O	M+	
559.2754	1	72437.35	C30H32F2N8O	(M+H)+	
560.2782	1	24304.75	C30H32F2N8O	(M+H)+	
561.2797	1	4693.79	C30H32F2N8O	(M+H)+	
562.2809	1	653.15	C30H32F2N8O	(M+H)+	
581.2578	1	674.76	C30H32F2N8O	(M+Na)+	
582.2601	1	194.23	C30H32F2N8O	(M+Na)+	
597.2306	1	441.14	C30H32F2N8O	(M+K)+	
280.1427	2	1530.98	C30H32F2N8O	(M+2H)+2	7.22
280.6426	2	558.26	C30H32F2N8O	(M+2H)+2	2.04
558.2623	1	438.14	C30H32F2N8O	M+	-7
559.2754	1	72437.35	C30H32F2N8O	(M+H)+	2.57
560.2782	1	24304.75	C30H32F2N8O	(M+H)+	2.41
561.2797	1	4693.79	C30H32F2N8O	(M+H)+	0.03
562.2809	1	653.15	C30H32F2N8O	(M+H)+	-2.67
581.2578	1	674.76	C30H32F2N8O	(M+Na)+	3.18
582.2601	1	194.23	C30H32F2N8O	(M+Na)+	2.15
597.2306	1	441.14	C30H32F2N8O	(M+K)+	1.15

--- End Of Report ---

Compound 7 ^{1}H NMR and ^{13}C NMR

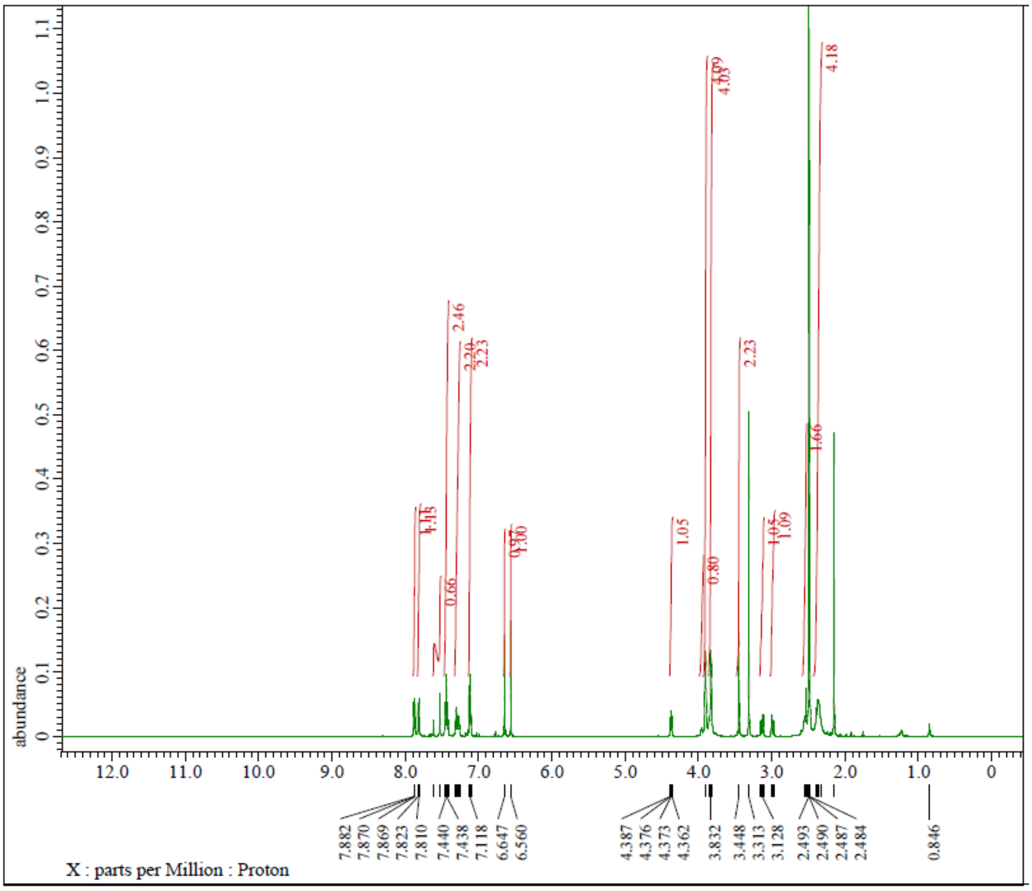

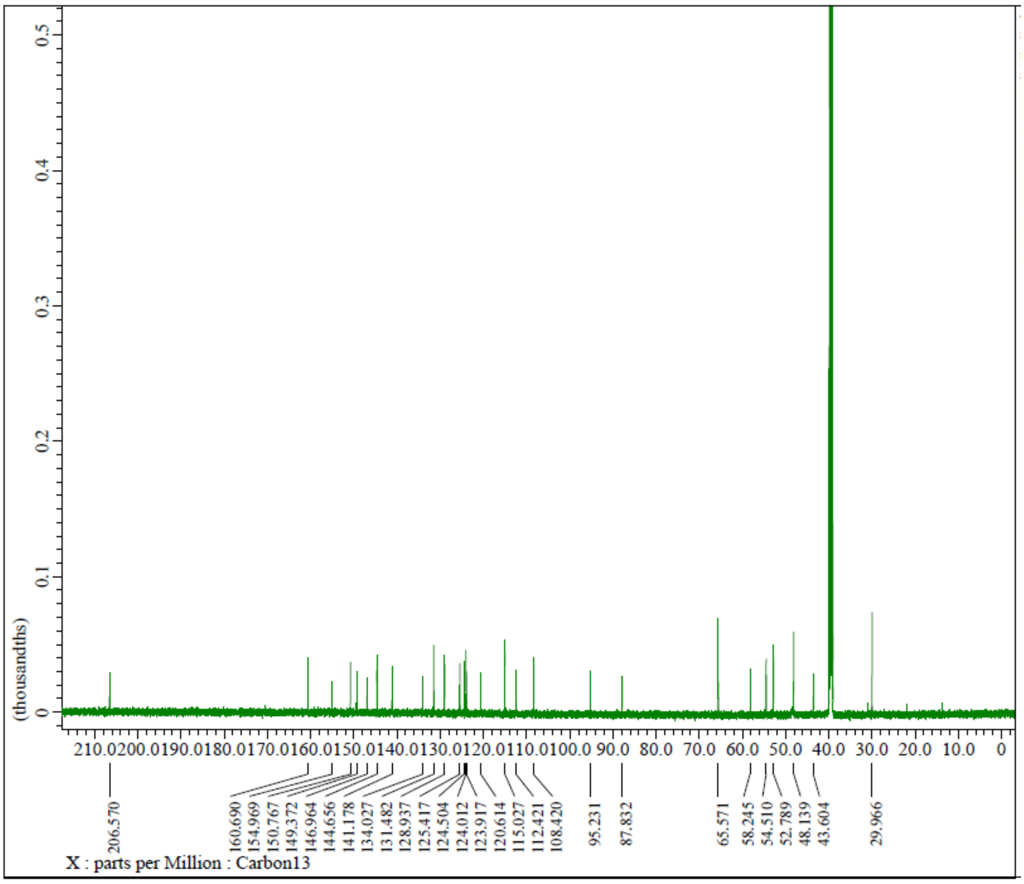

Compound 7 HRMS

Name	*Obs. m/z*	Obs. RT	Obs. Mass	DB RT	DB Formula	DB Mass	DB Mass Error	Tgt RT	Tgt Formula	Tgt Mass	Tgt Mass Error	RT Diff.	Find Cpds Algorith
	288.129	1.281	576.2573						C30 H31 F3 N8 O	576.2573	-0.05		Find by Formula

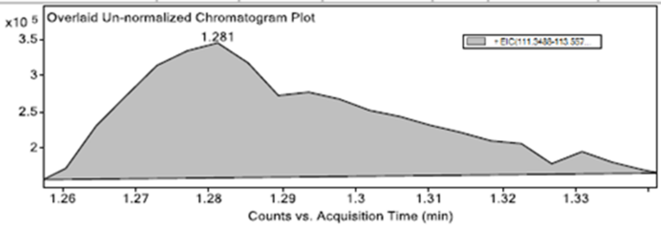

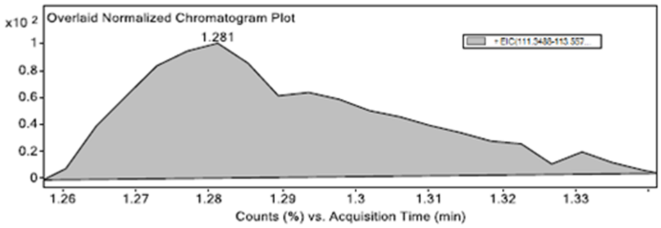

MS Spectrum Peak List

Obs. m/z	Charge	Abund	Formula	Ion/Isotope	Tgt Mass Error (ppm)
187.0937	3	66.4	C30H31F3N8O	(M+3H)+3[-H2O]	
288.129	2	1890.42	C30H31F3N8O	M+2	
288.6297	2	719.73	C30H31F3N8O	M+2	
289.1321	2	84.28	C30H31F3N8O	M+2	
299.11	2	273.47	C30H31F3N8O	(M+H+K)+2[-H2O]	
576.2519	1	1124.99	C30H31F3N8O	M+	
577.2591	1	227.25	C30H31F3N8O	M+	
187.0937	3	66.4	C30H31F3N8O	(M+3H)+3[-H2O]	22.11
288.129	2	1890.42	C30H31F3N8O	M+2	3.16
288.6297	2	719.73	C30H31F3N8O	M+2	0.58
289.1321	2	84.28	C30H31F3N8O	M+2	4.18
299.11	2	273.47	C30H31F3N8O	(M+H+K)+2[-H2O]	4.64
575.2501		3252.42			
576.2519	1	1124.99	C30H31F3N8O	M+	-8.43
577.2591	1	227.25	C30H31F3N8O	M+	-0.91

--- End Of Report ---

Compound 8 ^{1}H NMR and ^{13}C NMR

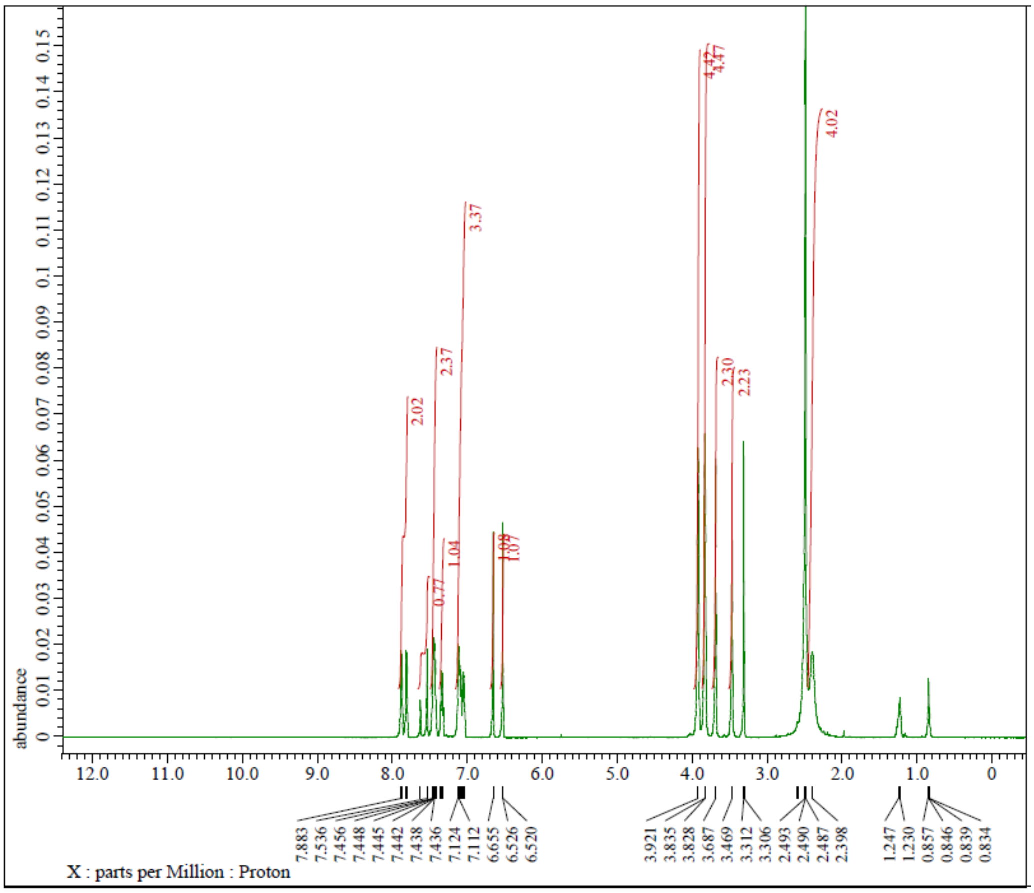

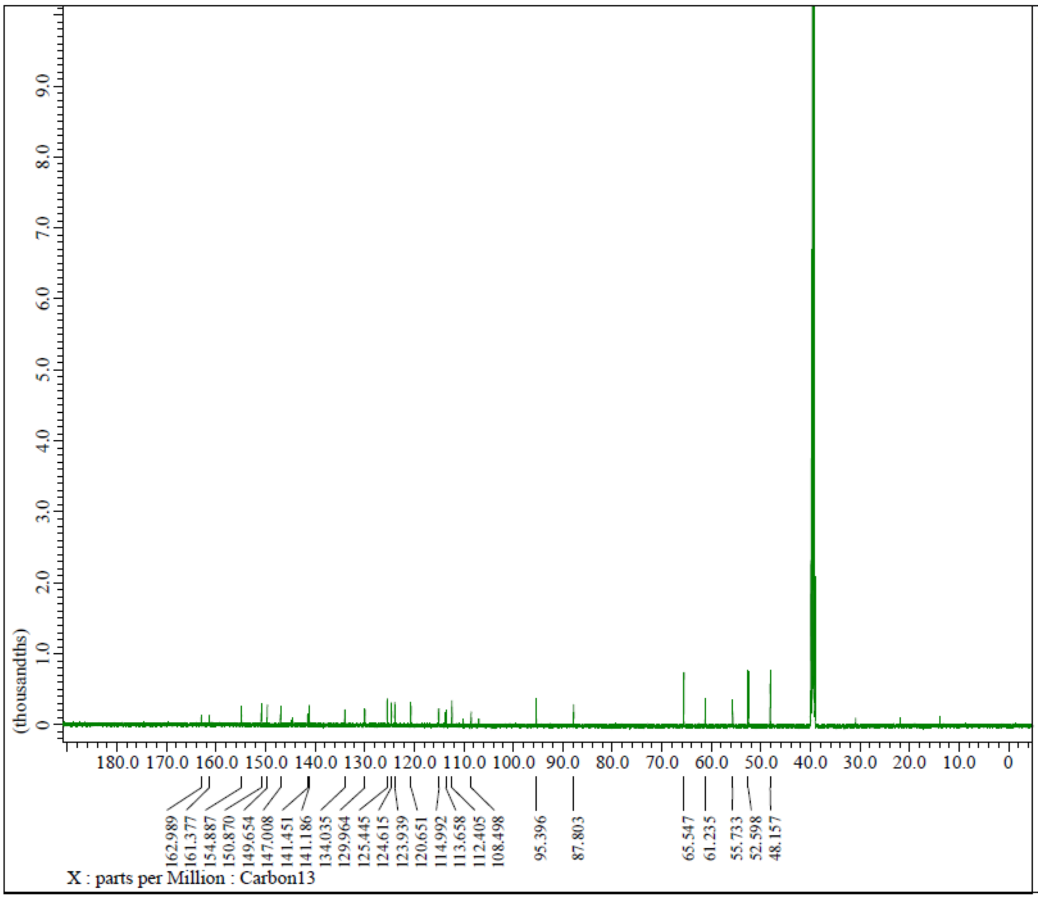

Compound 8 HRMS

Name	*Obs. m/z*	Obs. RT	Obs. Mass	DB RT	DB Formula	DB Mass	DB Mass Error	Tgt RT	Tgt Formula	Tgt Mass	Tgt Mass Error	RT Diff.	Find Cpds Algorith
	289.1371	1.277	576.2589						C30 H31 F3 N8 O	576.2573	2.74		Find by Formula

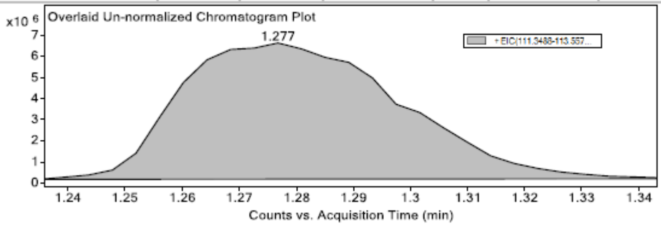

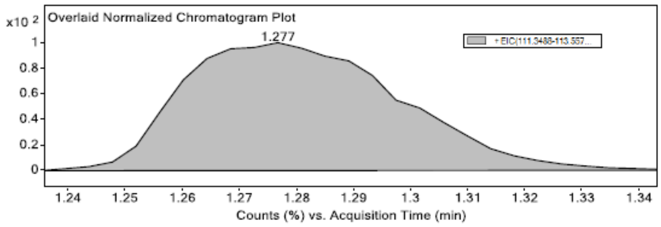

MS Spectrum Peak List

Obs. m/z	Charge	Abund	Formula	Ion/Isotope	Tgt Mass Error (ppm)
289.1371	2	3453.42	C30H31F3N8O	(M+2H)+2	
289.6383	2	1308.57	C30H31F3N8O	(M+2H)+2	
290.1397	2	224.68	C30H31F3N8O	(M+2H)+2	
576.2527	1	1496.31	C30H31F3N8O	M+	
577.2662	1	112540.48	C30H31F3N8O	(M+H)+	
578.2688	1	39327.02	C30H31F3N8O	(M+H)+	
579.2736	1	8483.74	C30H31F3N8O	(M+H)+	
599.2458	1	1163.36	C30H31F3N8O	(M+Na)+	
600.2491	1	475.19	C30H31F3N8O	(M+Na)+	
615.2252	1	538.34	C30H31F3N8O	(M+K)+	
289.1371	2	3453.42	C30H31F3N8O	(M+2H)+2	3.93
289.6383	2	1308.57	C30H31F3N8O	(M+2H)+2	3.16
290.1397	2	224.68	C30H31F3N8O	(M+2H)+2	3.12
576.2527	1	1496.31	C30H31F3N8O	M+	-6.93
577.2662	1	112540.48	C30H31F3N8O	(M+H)+	2.75
578.2688	1	39327.02	C30H31F3N8O	(M+H)+	2.39
579.2736	1	8483.74	C30H31F3N8O	(M+H)+	5.78
599.2458	1	1163.36	C30H31F3N8O	(M+Na)+	-1.11
600.2491	1	475.19	C30H31F3N8O	(M+Na)+	-0.53
615.2252	1	538.34	C30H31F3N8O	(M+K)+	7.77

--- End Of Report ---

Compound 9 ^{1}H NMR and ^{13}C NMR

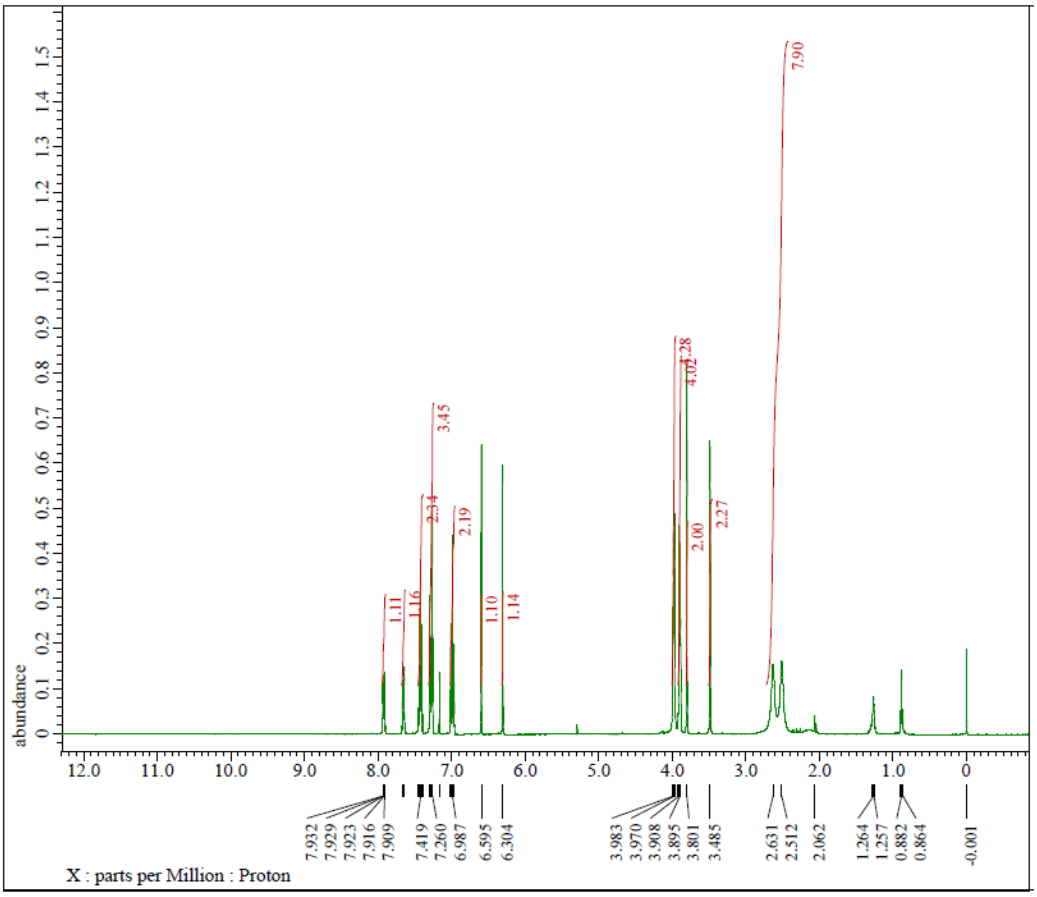

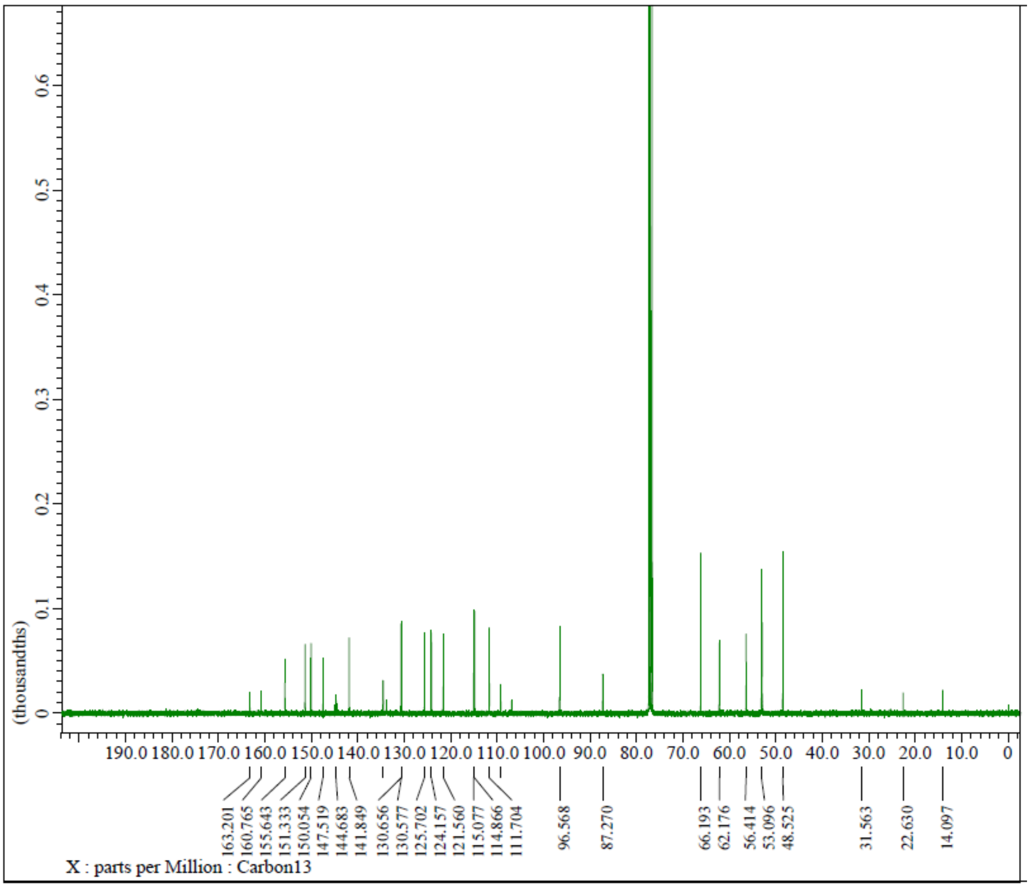

Compound 9 HRMS

Name	Obs. m/z	Obs. RT	Obs. Mass	DB RT	DB Formula	DB Mass	DB Mass Error	Tgt RT	Tgt Formula	Tgt Mass	Tgt Mass Error	RT Diff.	Find Cpds Algorith
	289.1367	1.269	576.2588						C30 H31 F3 N8 O	576.2573	2.57		Find by Formula

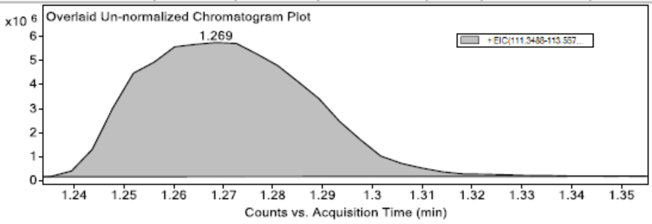

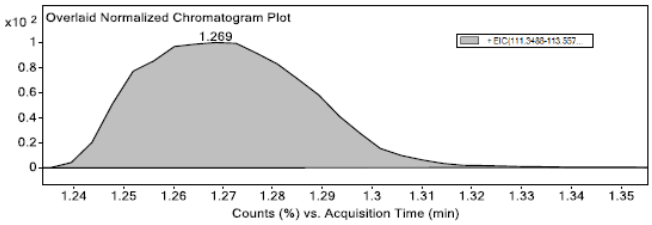

MS Spectrum Peak List

Obs. m/z	Charge	Abund	Formula	Ion/Isotope	Tgt Mass Error (ppm)
289.1367	2	2199.77	C30H31F3N8O	(M+2H)+2	
289.6382	2	788.68	C30H31F3N8O	(M+2H)+2	
576.2518	1	1086.32	C30H31F3N8O	M+	
577.2662	1	78180.23	C30H31F3N8O	(M+H)+	
578.269	1	26799.06	C30H31F3N8O	(M+H)+	
579.2708	1	4449.14	C30H31F3N8O	(M+H)+	
580.2718	1	689.42	C30H31F3N8O	(M+H)+	
599.2478	1	2053.6	C30H31F3N8O	(M+Na)+	
600.2499	1	749.33	C30H31F3N8O	(M+Na)+	
615.2209	1	1033.59	C30H31F3N8O	(M+K)+	
289.1367	2	2199.77	C30H31F3N8O	(M+2H)+2	2.8
289.6382	2	788.68	C30H31F3N8O	(M+2H)+2	3.04
576.2518	1	1086.32	C30H31F3N8O	M+	-8.56
577.2662	1	78180.23	C30H31F3N8O	(M+H)+	2.87
578.269	1	26799.06	C30H31F3N8O	(M+H)+	2.64
579.2708	1	4449.14	C30H31F3N8O	(M+H)+	0.88
580.2718	1	689.42	C30H31F3N8O	(M+H)+	-2.1
599.2478	1	2053.6	C30H31F3N8O	(M+Na)+	2.15
600.2499	1	749.33	C30H31F3N8O	(M+Na)+	0.86
615.2209	1	1033.59	C30H31F3N8O	(M+K)+	0.72

--- End Of Report ---

Compound 10 ^{1}H NMR and ^{13}C NMR

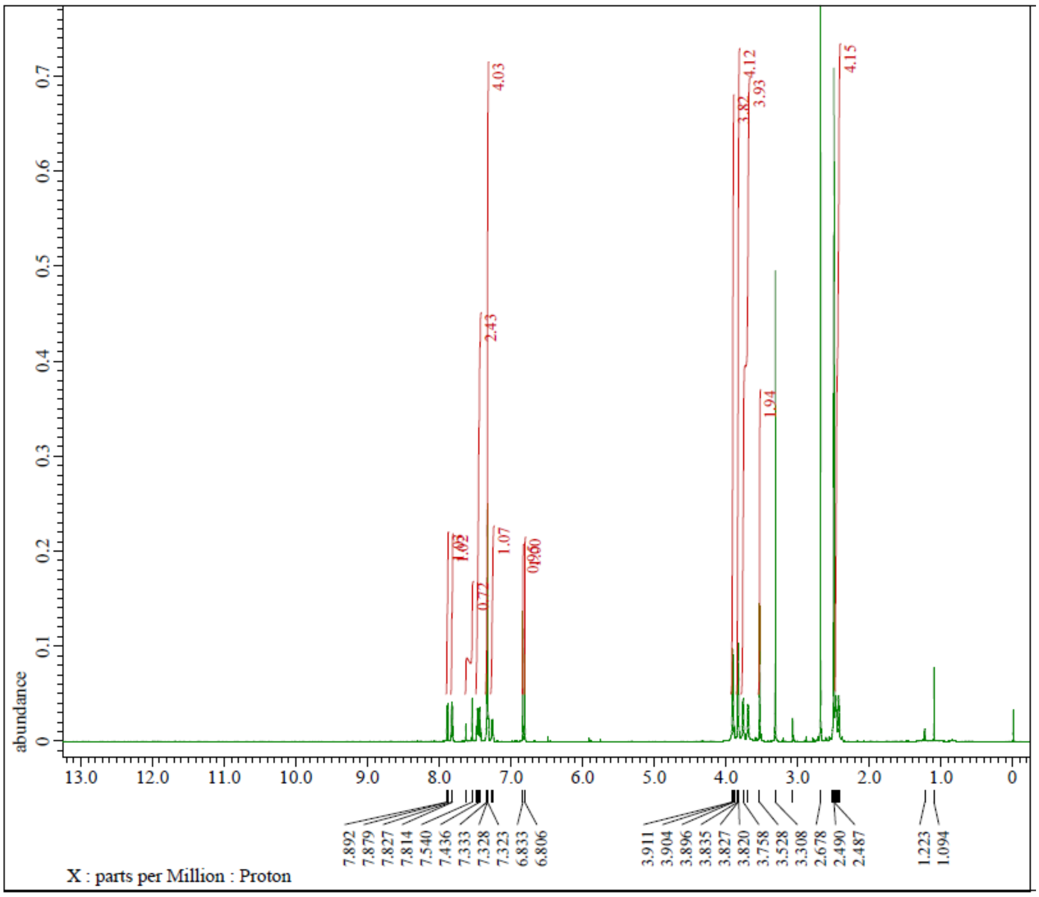

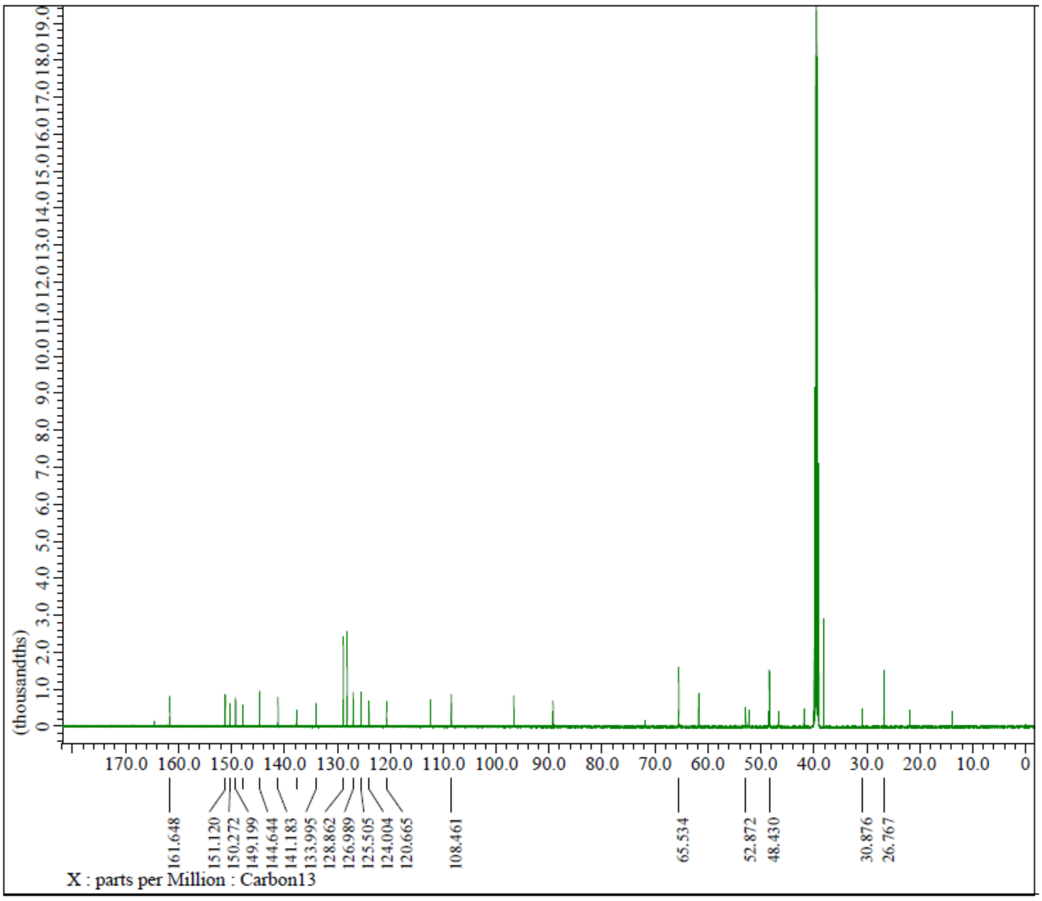

Compound 10 HRMS

Name	*Obs. m/z*	Obs. RT	Obs. Mass	DB RT	DB Formula	DB Mass	DB Mass Error	Tgt RT	Tgt Formula	Tgt Mass	Tgt Mass Error	RT Diff.	Find Cpds Algorith
	287.1309	1.242	572.2477						C30 H30 F2 N8 O2	572.246	2.93		Find by Formula

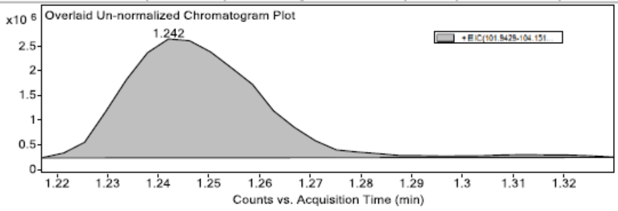

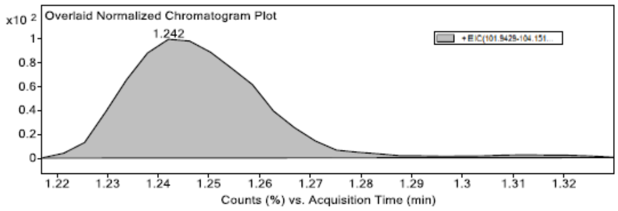

MS Spectrum Peak List

Obs. m/z	Charge	Abund	Formula	Ion/Isotope	Tgt Mass Error (ppm)
287.1309	2	41834.28	C30H30F2N8O2	(M+2H)+2	
287.6326	2	15865.28	C30H30F2N8O2	(M+2H)+2	
288.1343	2	3384.59	C30H30F2N8O2	(M+2H)+2	
573.2551	1	268057.69	C30H30F2N8O2	(M+H)+	
574.2581	1	87802.78	C30H30F2N8O2	(M+H)+	
575.2591	1	16343.6	C30H30F2N8O2	(M+H)+	
595.2362	1	18489.61	C30H30F2N8O2	(M+Na)+	
596.2394	1	5864.48	C30H30F2N8O2	(M+Na)+	
611.2102	1	11372.2	C30H30F2N8O2	(M+K)+	
612.212	1	3536.5	C30H30F2N8O2	(M+K)+	
287.1309	2	41834.28	C30H30F2N8O2	(M+2H)+2	2.08
287.6326	2	15865.28	C30H30F2N8O2	(M+2H)+2	3.14
288.1343	2	3384.59	C30H30F2N8O2	(M+2H)+2	4.33
573.2551	1	268057.69	C30H30F2N8O2	(M+H)+	3.27
574.2581	1	87802.78	C30H30F2N8O2	(M+H)+	3.46
575.2591	1	16343.6	C30H30F2N8O2	(M+H)+	0.35
595.2362	1	18489.61	C30H30F2N8O2	(M+Na)+	1.62
596.2394	1	5864.48	C30H30F2N8O2	(M+Na)+	2.24
611.2102	1	11372.2	C30H30F2N8O2	(M+K)+	1.66
612.212	1	3536.5	C30H30F2N8O2	(M+K)+	-0.05

--- End Of Report ---

Compound 11 ^{1}H NMR and ^{13}C NMR

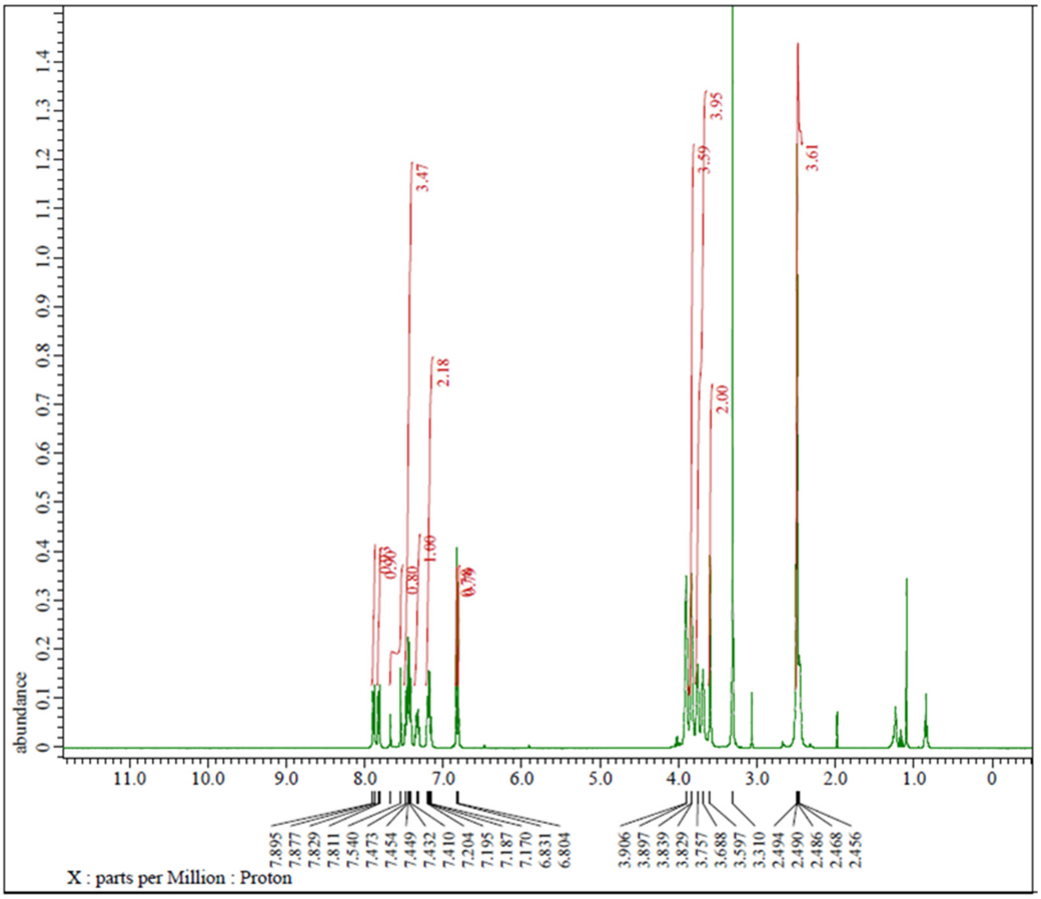

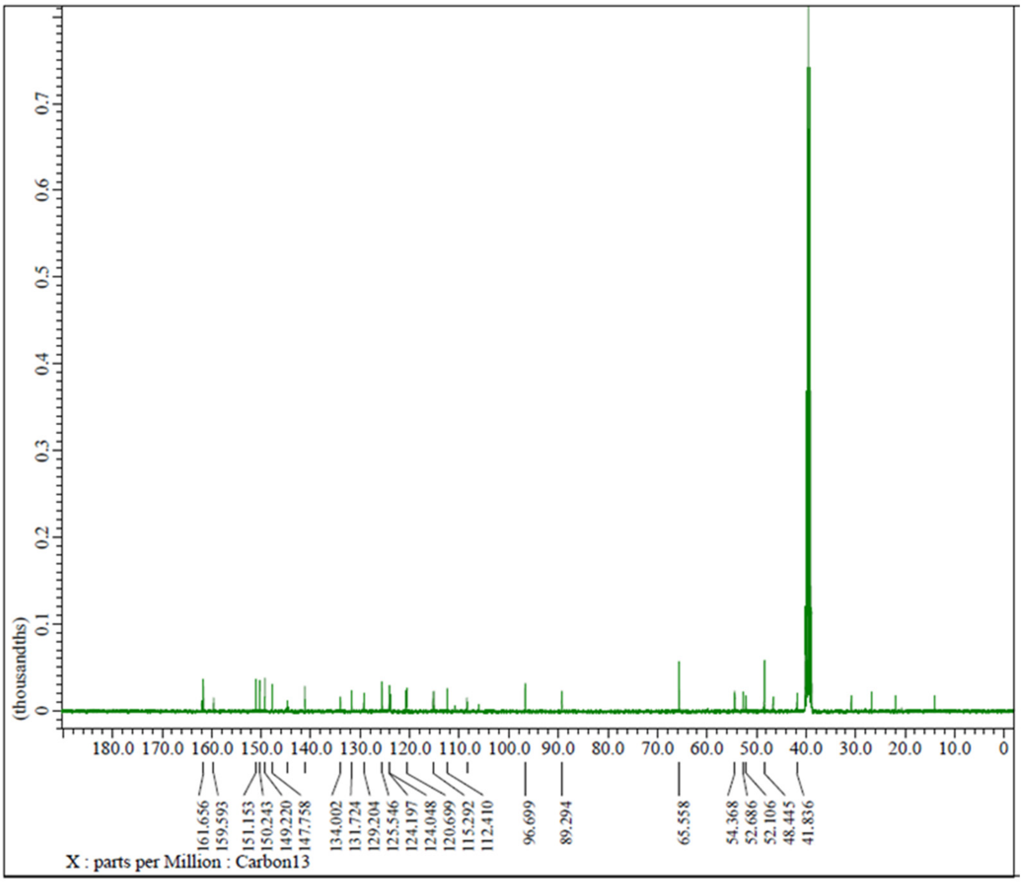

Compound 11 HRMS

Name	Obs. m/z	Obs. RT	Obs. Mass	DB RT	DB Formula	DB Mass	DB Mass Error	Tgt RT	Tgt Formula	Tgt Mass	Tgt Mass Error	RT Diff.	Find Cpds Algorith
	613.2261	1.252	590.2381						C30 H29 F3 N8 O2	590.2366	2.66		Find by Formula

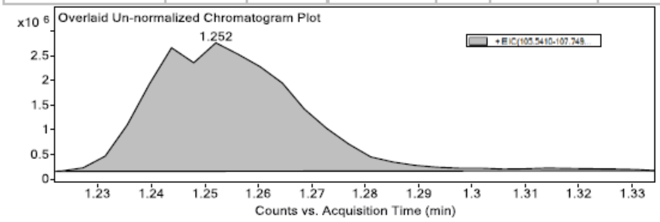

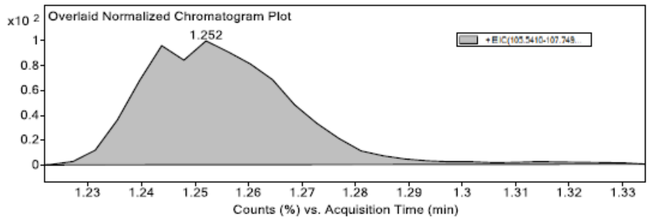

MS Spectrum Peak List

Obs. m/z	Charge	Abund	Formula	Ion/Isotope	Tgt Mass Error (ppm)
296.1262	2	35553	C30H29F3N8O2	(M+2H)+2	
296.6277	2	13484.65	C30H29F3N8O2	(M+2H)+2	
297.129	2	2209.65	C30H29F3N8O2	(M+2H)+2	
591.2456	1	236382.28	C30H29F3N8O2	(M+H)+	
592.2484	1	83000.14	C30H29F3N8O2	(M+H)+	
593.2504	1	14771.74	C30H29F3N8O2	(M+H)+	
613.2261	1	15137.5	C30H29F3N8O2	(M+Na)+	
614.2292	1	5721.4	C30H29F3N8O2	(M+Na)+	
629.2007	1	11055.59	C30H29F3N8O2	(M+K)+	
630.2018	1	4288.45	C30H29F3N8O2	(M+K)+	
296.1262	2	35553	C30H29F3N8O2	(M+2H)+2	2.14
296.6277	2	13484.65	C30H29F3N8O2	(M+2H)+2	2.36
297.129	2	2209.65	C30H29F3N8O2	(M+2H)+2	2.09
591.2456	1	236382.28	C30H29F3N8O2	(M+H)+	3
592.2484	1	83000.14	C30H29F3N8O2	(M+H)+	2.9
593.2504	1	14771.74	C30H29F3N8O2	(M+H)+	1.55
613.2261	1	15137.5	C30H29F3N8O2	(M+Na)+	0.57
614.2292	1	5721.4	C30H29F3N8O2	(M+Na)+	0.91
629.2007	1	11055.59	C30H29F3N8O2	(M+K)+	1.54
630.2018	1	4288.45	C30H29F3N8O2	(M+K)+	-1.23

--- End Of Report ---

Compound 12 ^{1}H NMR and ^{13}C NMR

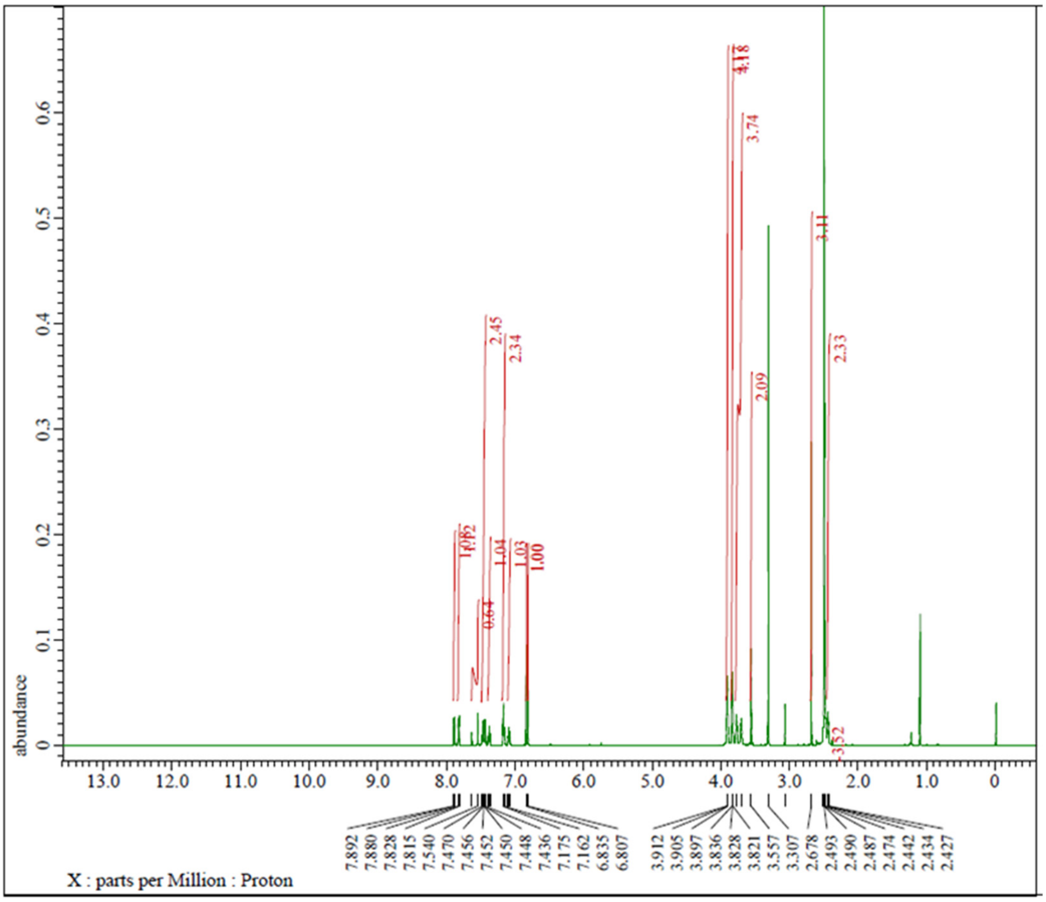

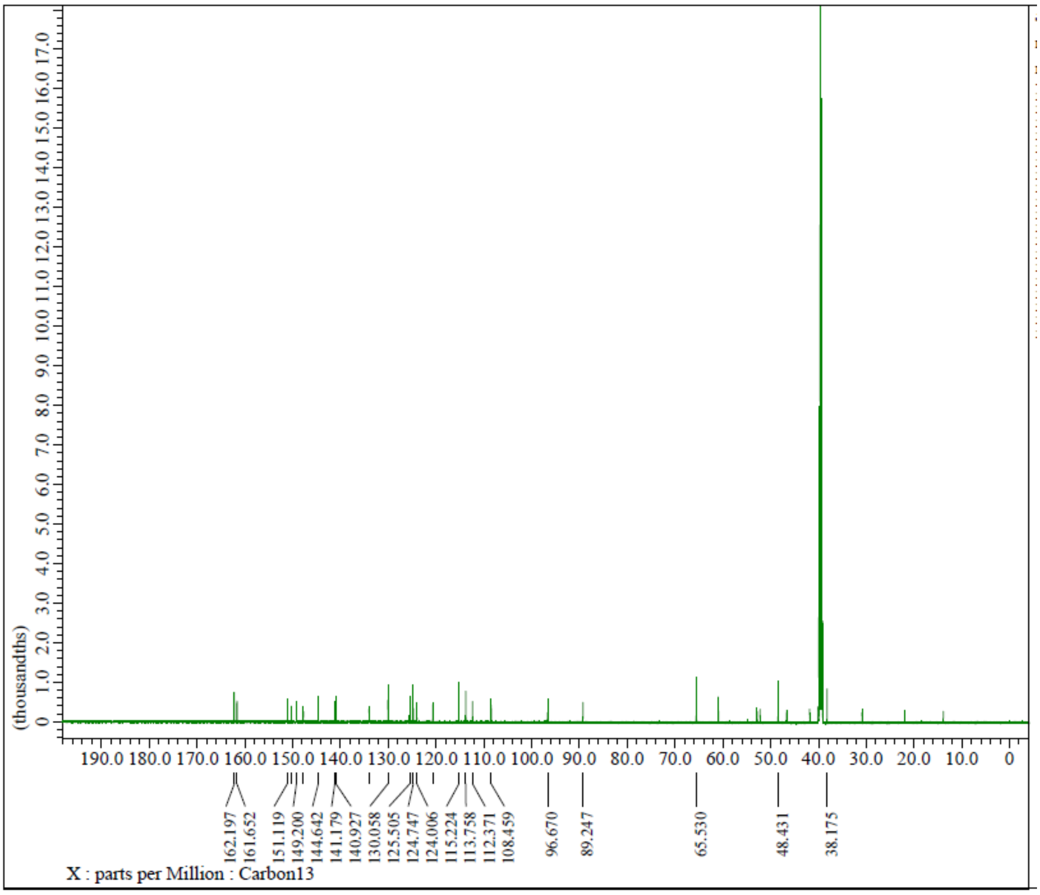

Compound 12 HRMS

Name	*Obs. m/z*	Obs. RT	Obs. Mass	DB RT	DB Formula	DB Mass	DB Mass Error	Tgt RT	Tgt Formula	Tgt Mass	Tgt Mass Error	RT Diff.	Find Cpds Algorith
**302-612-01**	613.2264	1.261	590.238						C30 H29 F3 N8 O2	590.2366	2.51		Find by Formula

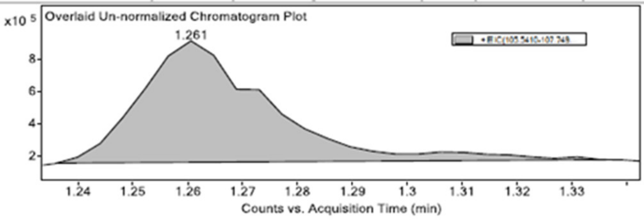

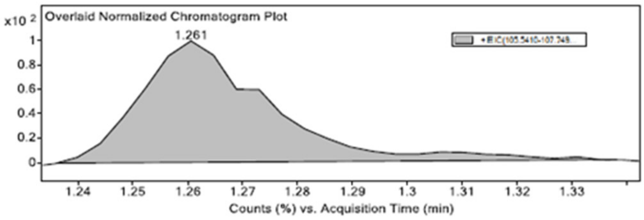

MS Spectrum Peak List

Obs. m/z	Charge	Abund	Formula	Ion/Isotope	Tgt Mass Error (ppm)
296.1264	2	49882.54	C30H29F3N8O2	(M+2H)+2	
296.6278	2	18123.12	C30H29F3N8O2	(M+2H)+2	
297.1291	2	3480.89	C30H29F3N8O2	(M+2H)+2	
591.2454	1	213606.42	C30H29F3N8O2	(M+H)+	
592.2484	1	70680.39	C30H29F3N8O2	(M+H)+	
593.2501	1	12701.99	C30H29F3N8O2	(M+H)+	
613.2264	1	23715.41	C30H29F3N8O2	(M+Na)+	
614.2296	1	8195.56	C30H29F3N8O2	(M+Na)+	
629.2001	1	13863.15	C30H29F3N8O2	(M+K)+	
630.2036	1	4673.35	C30H29F3N8O2	(M+K)+	
296.1264	2	49882.54	C30H29F3N8O2	(M+2H)+2	2.99
296.6278	2	18123.12	C30H29F3N8O2	(M+2H)+2	2.7
297.1291	2	3480.89	C30H29F3N8O2	(M+2H)+2	2.49
591.2454	1	213606.42	C30H29F3N8O2	(M+H)+	2.72
592.2484	1	70680.39	C30H29F3N8O2	(M+H)+	2.86
593.2501	1	12701.99	C30H29F3N8O2	(M+H)+	1.14
613.2264	1	23715.41	C30H29F3N8O2	(M+Na)+	1.02
614.2296	1	8195.56	C30H29F3N8O2	(M+Na)+	1.61
629.2001	1	13863.15	C30H29F3N8O2	(M+K)+	0.69
630.2036	1	4673.35	C30H29F3N8O2	(M+K)+	1.54

--- End Of Report ---

Compound 13 ^{1}H NMR and ^{13}C NMR

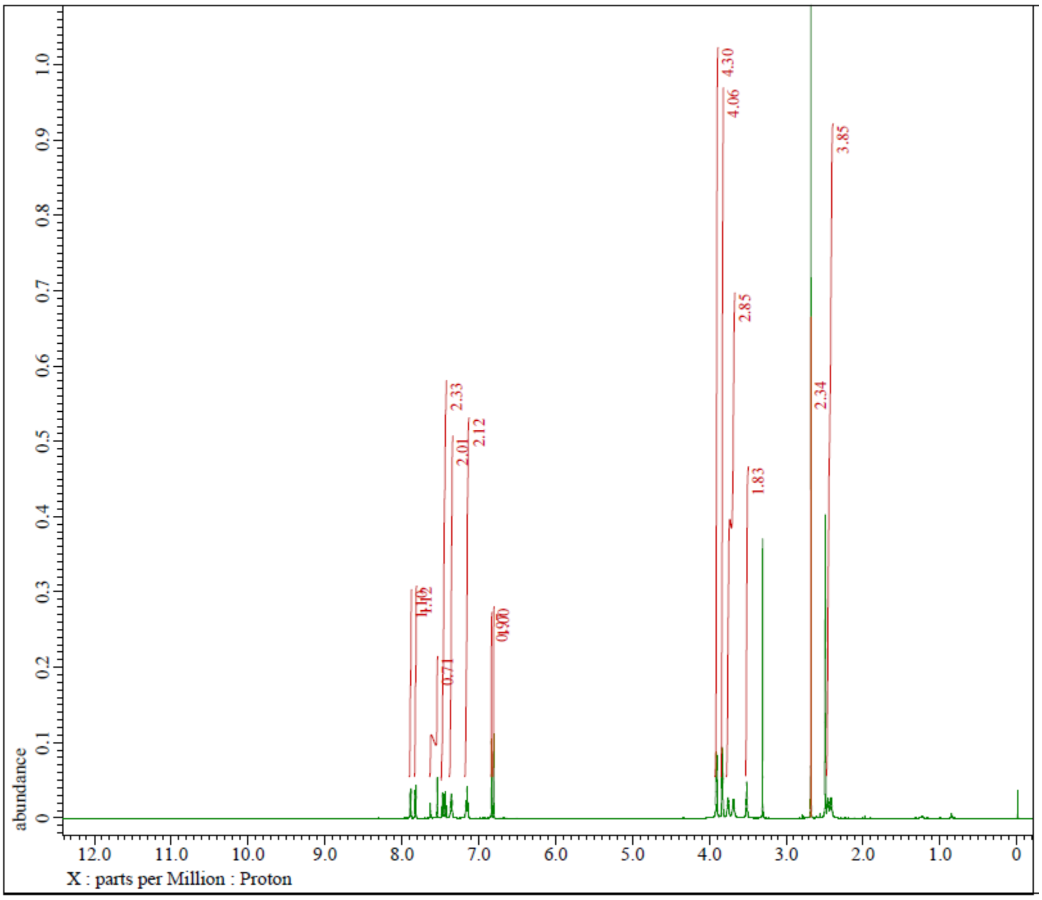

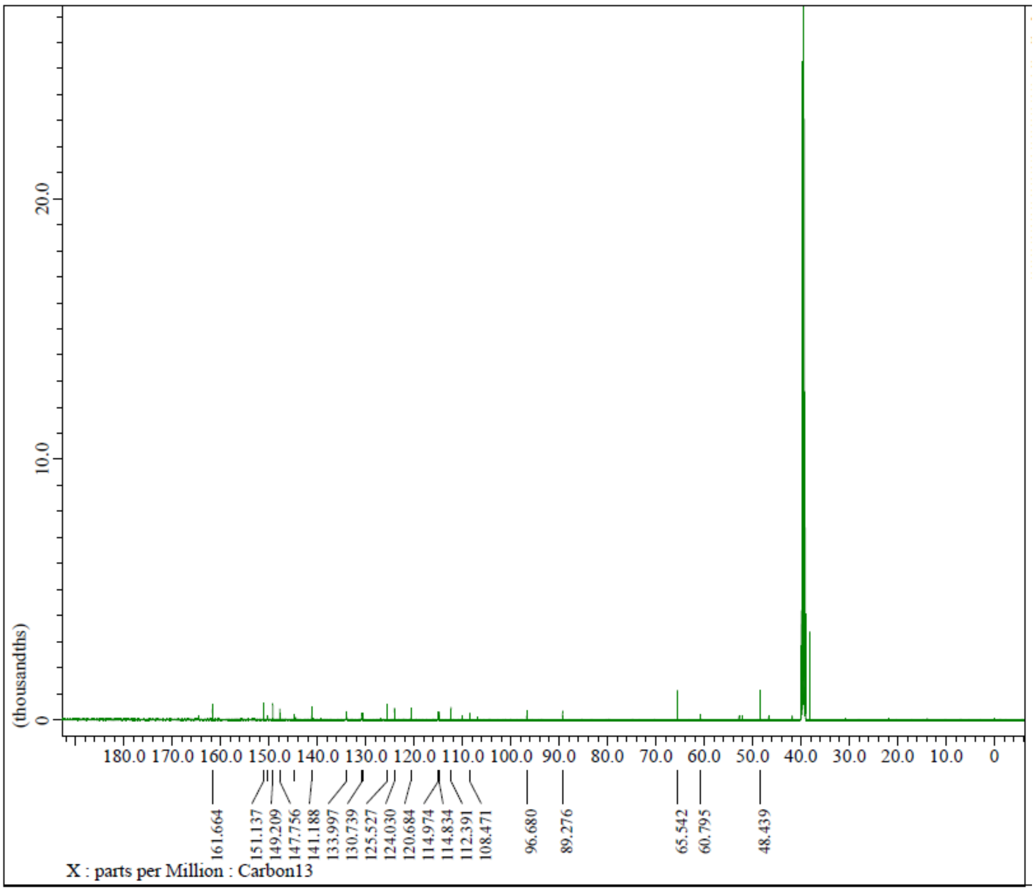

Compound 13 HRMS

Name	*Obs. m/z*	Obs. RT	Obs. Mass	DB RT	DB Formula	DB Mass	DB Mass Error	Tgt RT	Tgt Formula	Tgt Mass	Tgt Mass Error	RT Diff.	Find Cpds Algorith
	613.2269	1.253	590.238						C30 H29 F3 N8 O2	590.2366	2.46		Find by Formula

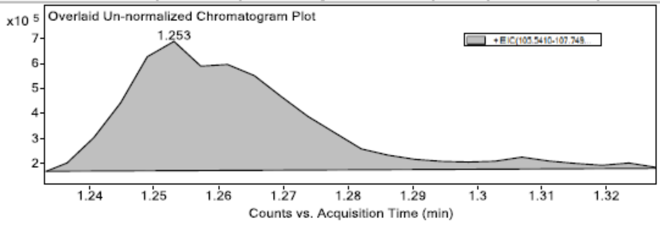

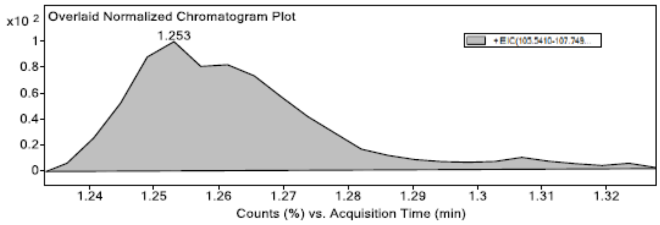

MS Spectrum Peak List

Obs. m/z	Charge	Abund	Formula	Ion/Isotope	Tgt Mass Error (ppm)
296.1265	2	25369.7	C30H29F3N8O2	(M+2H)+2	
296.6281	2	8685.4	C30H29F3N8O2	(M+2H)+2	
590.2317	1	6814.01	C30H29F3N8O2	M+	
591.2455	1	156091.31	C30H29F3N8O2	(M+H)+	
592.2482	1	55143.44	C30H29F3N8O2	(M+H)+	
593.2506	1	9649.6	C30H29F3N8O2	(M+H)+	
613.2269	1	23424.83	C30H29F3N8O2	(M+Na)+	
614.2291	1	8010.62	C30H29F3N8O2	(M+Na)+	
629.2012	1	15108.07	C30H29F3N8O2	(M+K)+	
630.2039	1	5253.17	C30H29F3N8O2	(M+K)+	
296.1265	2	25369.7	C30H29F3N8O2	(M+2H)+2	3.29
296.6281	2	8685.4	C30H29F3N8O2	(M+2H)+2	3.6
590.2317	1	6814.01	C30H29F3N8O2	M+	-7.22
591.2455	1	156091.31	C30H29F3N8O2	(M+H)+	2.89
592.2482	1	55143.44	C30H29F3N8O2	(M+H)+	2.53
593.2506	1	9649.6	C30H29F3N8O2	(M+H)+	1.96
613.2269	1	23424.83	C30H29F3N8O2	(M+Na)+	1.79
614.2291	1	8010.62	C30H29F3N8O2	(M+Na)+	0.66
629.2012	1	15108.07	C30H29F3N8O2	(M+K)+	2.33
630.2039	1	5253.17	C30H29F3N8O2	(M+K)+	2.12

--- End Of Report ---

Synthesis of 5-(2-difluoromethylobenzimidazo-1-yl)pyrazolo[1,5-a]pyrimidine derivatives.

Synthesis of 5-(2-difluoromethylobenzimidazo-1-yl)pyrazolo[1,5-*a*]pyrimidine derivatives Stypik M. et.all, *Pharmaceuticals* **2022**, 15, 927; 949).

Synthesis of 5-(2-difluoromethylobenzimidazo-1-yl)pyrazolo[1,5-*a*]pyrimidine derivatives. Reagents and conditions: (**i**) 2-(difluoromethyl)-1*H*-benzimidazole, TEACl, K_2CO_3, DMA, 160 °C, 3 h, 89%; (**ii**) $LiAlH_4$, THF, 0 °C, 3 h, 89%, (**iii**) Dess–Martin periodinane, DMF, RT, 1 h, 78% or MnO_2, toluene:buthyl acetate, reflux 1.5 h, 68%; (**iv**) amine, sodium triacetoxyborohydride, DCM, 18 h, 38–93%; (**v**) LiOH, MeOH, H_2O, 98%; (**vi**) amine, HATU, TEA, RT, 2 h, 33–81% (Stypik M. et.all, *Pharmaceuticals* **2022**, 15, 927).

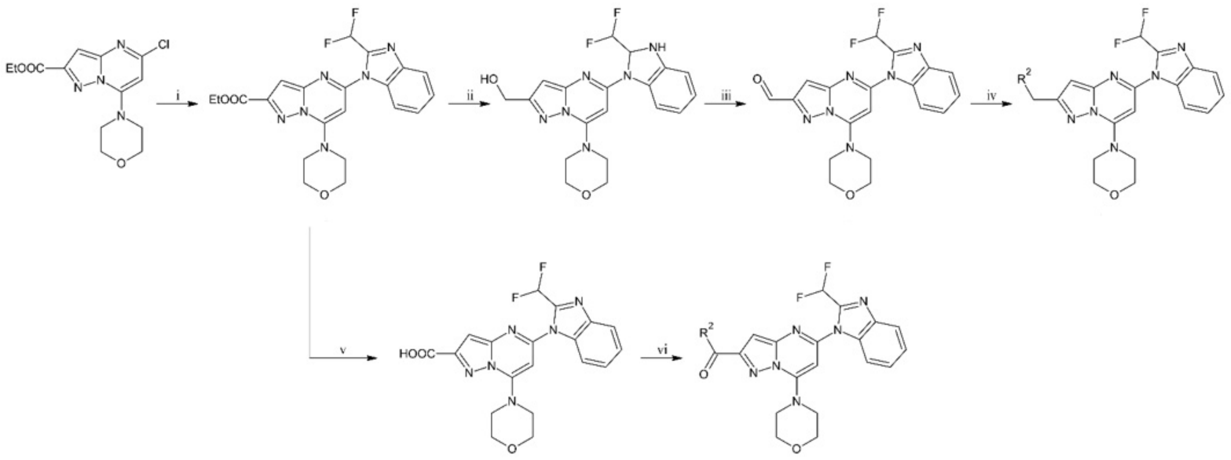

The model system of PI3Kδ with lipid bilayer

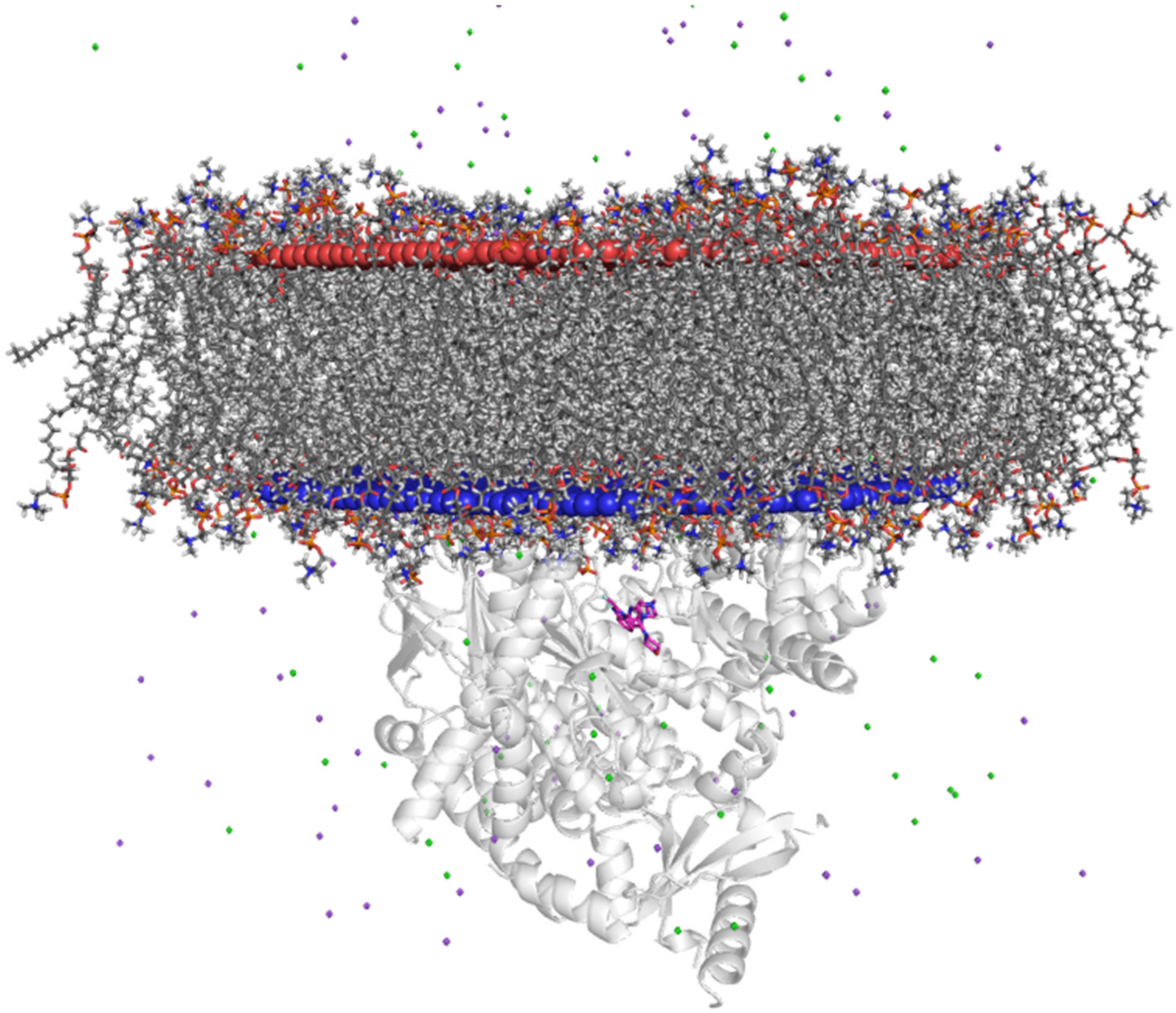

An example of a PI3Kδ kinase system with an inhibitor attached to a lipid bilayer.

The results of QPLD docking approach with different level of flexibility of protein.

Table S1. Influence of the fluorine atom(s) on PI3Kδ inhibition and the respective ΔG and ΔΔG[a] scores for each compound using QPLD docking approach to rigid conformation of PI3K*δ* kinase (PDB ID: 2WXL).

Compound	R[1]	R[2]	IC_{50} PI3Kδ [nM][b]	$\overline{\Delta G}$ [kcal/mol]	ΔΔG [kcal/mol]
**1**	**CH_3**	**H**	236	−71.7	–
**2 (CPL302415)**	**CHF_2**	**H**	18	−74.5	−2.8
**3**	**CF_3**	**H**	907	−73.2	−1.5
**4**	**CHF_2**	**Cl**	44	−68.9	5.5[c]
**5**	**CHF_2**	**Br**	50	−70.5	4.0[c]

[a]The interaction energy gain averaged by three ligand–receptor complexes of each derivative selected from the MD simulations.
[b]IC_{50} values were determined as the mean from two independent experiments.
[c]ΔΔG values calculated as a difference between a given derivative and its nonhalogenated (difluoromethyl)-1*H*-benzimidazole analog.

Table S2. Influence of the fluorine atom(s) on PI3Kδ inhibition and the respective ΔG and ΔΔG[a] scores for each compound using QPLD docking approach to rigid conformation of PI3K*δ* kinase (PDB ID: 2WXL).

Compound	X	R[3]	IC_{50} PI3K*δ* [nM][b]	$\overline{\Delta G}$ [kcal/mol]	ΔΔG [kcal/mol]
**6**	**CH_2**	**-**	118	−74.3	–
**7**	**CH_2**	***o*-F**	640	−70.5	3.9
**8**	**CH_2**	***m*-F**	751	−73.4	1.0
**9**	**CH_2**	***p*-F**	489	−75.0	−0.7
**10**	**CO**	**-**	275	−76.6	–
**11**	**CO**	***o*-F**	212	−69.5	7.2
**12**	**CO**	***m*-F**	92	−69.2	7.4
**13**	**CO**	***p*-F**	181	−70.4	6.3

[a]The interaction energy gain averaged by three ligand–receptor complexes of each derivative selected from the MD simulations.
[b]IC_{50} values were determined as the mean from two independent experiments.

Table S3. Influence of the fluorine atom(s) on PI3Kδ inhibition and the respective ΔG and ΔΔG[a] scores for each compound using QPLD docking approach to conformation of PI3Kδ kinase obtained in IFD stage.

Compound	R[1]	R[2]	IC_{50} PI3Kδ [nM][b]	$\overline{\Delta G}$ [kcal/mol]	ΔΔG [kcal/mol]
**1**	**CH_3**	**H**	236	−84.2	–
**2 (CPL302415)**	**CHF_2**	**H**	18	−83.4	0.8
**3**	**CF_3**	**H**	907	−84.8	–0.6
**4**	**CHF_2**	**Cl**	44	−85.2	–1.8[c]
**5**	**CHF_2**	**Br**	50	−84.3	–0.9[c]

[a]The interaction energy gain averaged by three ligand–receptor complexes of each derivative selected from the MD simulations.
[b]IC_{50} values were determined as the mean from two independent experiments.
[c]ΔΔG values calculated as a difference between a given derivative and its nonhalogenated (difluoromethyl)-1*H*-benzimidazole analog.

Table S4. Influence of the fluorine atom(s) on PI3Kδ inhibition and the respective ΔG and ΔΔG[a] scores for each compound using QPLD docking approach to rigid conformation of PI3Kδ kinase obtained in IFD stage.

Compound	X	R[3]	IC_{50} PI3Kδ [nM][b]	$\overline{\Delta G}$ [kcal/mol]	ΔΔG [kcal/mol]
**6**	**CH_2**	**-**	118	−89.6	–
**7**	**CH_2**	***o*-F**	640	−87.3	2.3
**8**	**CH_2**	***m*-F**	751	−88.9	0.7
**9**	**CH_2**	***p*-F**	489	−88.3	1.3
**10**	**CO**	**-**	275	−88.0	–
**11**	**CO**	***o*-F**	212	−88.6	−0.6
**12**	**CO**	***m*-F**	92	−86.0	2.0
**13**	**CO**	***p*-F**	181	−88.2	−0.1

[a]The interaction energy gain averaged by three ligand–receptor complexes of each derivative selected from the MD simulations.
[b]IC_{50} values were determined as the mean from two independent experiments.
